# From Displacement to Angle: Diamond‐Based 3D Rotation Sensing for High‐Precision Cellular Force Measurement

**DOI:** 10.1002/advs.76685

**Published:** 2026-07-20

**Authors:** Linjie Ma, Bicong Wang, Tai Nam Yip, Jiahua Zhang, Yicheng Wang, Luyao Zhang, Yong Hou, Yuan Lin, Zhiqin Chu

**Affiliations:** ^1^ Department of Electrical and Computer Engineering the University of Hong Kong Hong Kong SAR Hong Kong; ^2^ Department of Mechanical Engineering the University of Hong Kong Hong Kong SAR Hong Kong

**Keywords:** angle sensing, cellular forces, diamond‐based sensing, mechanobiology, traction force microscopy

## Abstract

Cellular traction forces are conventionally measured by tracking the displacement of beads or micropillars and converting them to force via mechanical models. Although widely used, these displacement‐based methods primarily report translational motion and, when based on the linear Euler–Bernoulli beam assumption, can suffer from geometric‐nonlinearity errors under non‐negligible deformation. Here we introduce an alternative approach: quantifying force by directly measuring pillar rotation angle rather than displacement, using fluorescent nanodiamonds (FNDs) as embedded 3D orientation markers. Specifically, by integrating optically detected magnetic resonance (ODMR) with laser polarization modulation (LPM), we determine the complete three‑dimensional orientation of FNDs attached to polydimethylsiloxane (PDMS) micropillars with sub‑degree precision (∼0.5°). This angle‑based measurement framework enables force reconstruction from pillar rotation and provides a robust analytical readout for stocky beams and large deformations. Finite‑element simulations demonstrate that our method reduces force estimation errors by at least 10% compared to linear displacement‑based approaches. Moreover, we successfully capture three‐dimensional pillar deformations, including bending and in‐plane rotation, that are inaccessible to the conventional displacement‑only method. Taken together, our work establishes diamond‑based angular force microscopy as a high‑precision platform for mechanobiology.

## Graphical abstract

Cellular forces are traditionally inferred from displacement measurements, which lack rotational information and are constrained by linear model assumptions. This work replaces displacement with rotational angle for the fundamental mechanical readout. Using diamond quantum sensing, pillar rotations are directly measured, enabling accurate three‐dimensional cellular force quantification and extending traction force microscopy toward angle‐resolved biomechanical sensing.

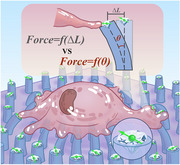

## 1 Introduction

**FIGURE 1:**
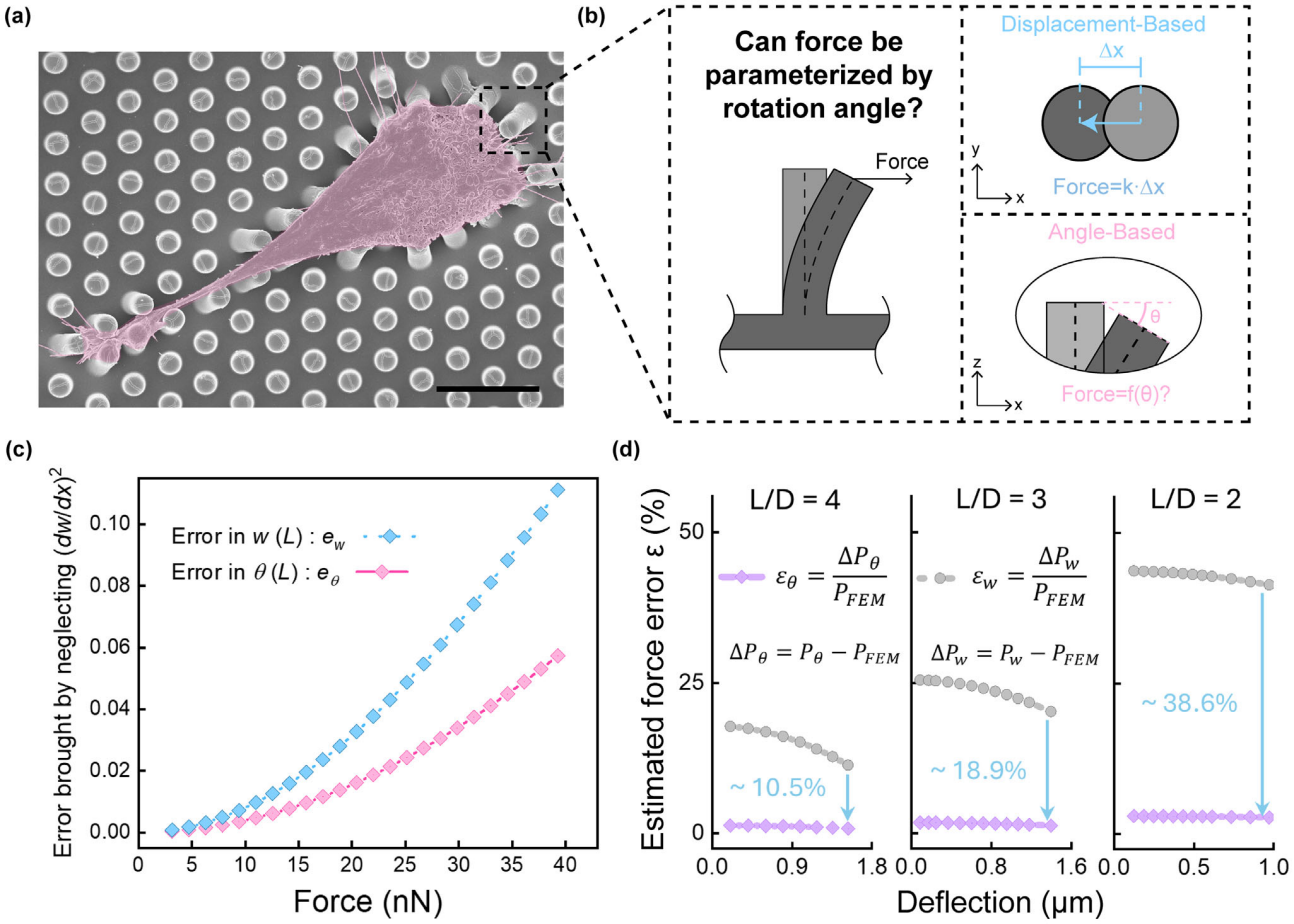
Rotation angle as an alternative high‐accuracy mechanical readout for cellular force measurement. (a) SEM image of bent PDMS micropillars caused by cell traction force. The scale bar is 10 µm. (b) Comparison of the traditional displacement‐based method and the newly proposed angle‐based method. (c) Errors induced by 1 + (d*w*/d*x*)^2^ ≈ 1 within the framework of Euler‐Bernoulli beam theory. (d) The errors in estimated forces with pillar length *L* of 6 µm and diameter *D* of 1.5 µm, 2 µm, and 3 µm. *P_w_* and *P*_θ_ represent the force estimated from pillar deflection and rotation angle, respectively, and *P_FEM_* represents the force calculated from finite element simulations.

Cells generate mechanical forces on their surrounding matrix to regulate crucial cellular activities, such as spreading, migration, division, and differentiation [1, 2, 3, 4, 5, 6, 7, 8]. Accurate measurement of cell–substrate forces is therefore essential for understanding how cells sense and respond to their mechanical microenvironment [9, 10, 11], and for identifying mechanical biomarkers relevant to disease mechanobiology [12]. This need has motivated the development of a variety of traction force measurement platforms [13]. Conventional traction force measurement methods, including bead tracking in gels [14, 15, 16] and micropillar arrays [17], infer cellular forces from the measured displacements of markers (i.e., beads and micropillars) using simplified mechanical models. These displacement‐based approaches have been fundamental for analyzing cell mechanics, with advances in super‐resolution imaging and localization algorithms improving their accuracy, spatial resolution, and speed. However, many conventional implementations assume small and geometry‐preserving deformations, which may not always hold under cellular loading (Figure 1a). Although finite element methods (FEM) and nonlinear beam models can address these effects, their practical use is often complex and computationally demanding, motivating a simpler analytical solution.

Rather than seeking further improvements within the same translational measurement framework, we ask whether a different mechanical observable can provide a more informative and robust readout of cellular forces (Figure 1b). In displacement‐based methods, mechanical sensors are effectively treated as point‐like probes, whose motion is reduced to pure translation, leaving rotational degrees of freedom unmeasured even though they are intrinsically coupled to how forces are transmitted [18]. For example, in standard pillar‐based traction force microscopy, each polydimethylsiloxane (PDMS) post is modeled as an independent cantilever whose lateral force is inferred solely from tip displacement via a linear spring calibration *P*  =  3*EI*δ/*L* ^3^, derived from small‐deflection Euler–Bernoulli beam theory [19]. Yet any bending of a pillar necessarily produces not only a lateral deflection of the tip but also a finite tilt of the pillar axis in the vertical (x–z) plane. Conventional analyses use only the translational component δ and discard the tilting component. In addition, micropillars fabricated from PDMS, a material commonly used in mechanobiology, exhibit low bending stiffness. Consequently, cellular forces may induce relatively large deformations at the pillar top, thereby compromising the applicability of the linear force–displacement relationship. These limitations motivate an angle‐based readout in which the bending‐induced rotation angle replaces translational displacement for mechanical measurement. This approach becomes particularly advantageous under large pillar bending, where conventional displacement‐based models become unreliable.

To realize such an angle‑based readout, we need a method that can report the full three‑dimensional orientation of nanoscale markers on pillar tops with sub‑degree precision under diffraction‑limited imaging. Recently, diamond‐based quantum sensors have emerged as powerful tools for tracking multidimensional motions in biological systems [20, 21]. The negatively charged nitrogen–vacancy center (NV^−^ center, hereafter abbreviated as NV center) [22] in diamond acts as a built‐in sensor whose spin resonance is highly sensitive to the relative orientation of external magnetic field and NV center axis [23, 24, 25], enabling off‐axis rotation detection via optically detected magnetic resonance (ODMR, angular resolution∽0.5° [26]), while its fluorescence is polarization‐dependent [27, 28], allowing in‐plane rotation tracking via laser polarization modulation (LPM, angular resolution∽2–3° [18]). Using these methods, NV centers in diamond have been successfully used as orientation markers to measure nanoscale substrate deformation in force fields [18, 26]. However, initial studies primarily measured in‐plane rotation of FNDs in cell traction fields, providing only a two‐dimensional angular projection and preventing direct force quantification from rotation alone. Extending this capability to complete three‐dimensional orientation sensing, which is essential for resolving multidimensional forces and establishing robust angle‐to‐force calibration, remains challenging.

Here, we develop a hybrid ODMR‐LPM method that determines the complete three‐dimensional orientation of FNDs attached to PDMS micropillars with sub‐degree precision (≈0.5° for out‐of‐plane rotation and 3° for in‐plane rotation). By directly measuring the bending‐induced rotation angle, we establish an angle‐based force calibration framework that goes beyond translational motion and reduces the linearity constraints of conventional displacement tracking. An analytical treatment based on the exact differential equation governing the deflection of micropillars (irrespective of their aspect ratio) yields a relationship between the measured angle and the applied force. Finite‐element simulations demonstrate that this approach reduces force estimation errors by at least 10% compared to the basic traditional displacement‐based model, with the largest improvements occurring for low‐aspect‐ratio pillars where geometric nonlinearity is most pronounced. We further validate the 3D rotational tracking capability by using bulk diamond calibration standards, confirming an angular precision of ∼0.5° for out‐of‐plane rotations. Finally, we apply this platform to living cells and successfully resolve their traction fields.

## 2 Results and Discussion

### 2.1 Theoretical Validation of Angle as a Mechanical Readout in Pillar‐Based Traction Force Microscopy

The classical displacement‐based force estimation relies on the Euler‐Bernoulli beam theory, which assumes deformations are relatively small so that the cross‐section of the beam remains a plane after deflection. For a cantilever beam (Figure S1), the relationship between the concentrated force *P* at the free end and deformation (*θ* and *w* for rotational angle and deflection, respectively) can be described by a differential equation [29]:

(1) $$\frac{d^{2}w}{dx^{2}}\;\frac{1}{{\left(1+{\left(\frac{dw}{dx}\right)}^{2}\right)}^{\frac{3}{2}}}=\frac{d\theta }{dx}\frac{1}{\sqrt{1+{\left(\frac{dw}{dx}\right)}^{2}}}=\frac{P\left(L-x\right)}{EI}$$

where *E* represents the Young's modulus, *I* denotes the inertia moment of the beam cross‐section. Based on the small deformation assumption, the terms of (*dw*/*dx*)^2^ in the denominators of Equation (1) can be disregarded, leading to:

(2) $$w_{EB}\left(L\right)=\frac{PL^{3}}{3EI}\;,\;\theta_{EB}\left(L\right)=\frac{PL^{2}}{2EI}$$

The deviations incurred at the free end of the beam due to neglecting the (*dw*/*dx*)^2^ term can be estimated by (see Supporting Materials for details):

(3) $$e_{\theta }=\left|\frac{\theta_{EB}-\theta_{IF}}{\theta_{IF}}\right|\;\approx \frac{1}{1+\frac{24E^{2}I^{2}}{P^{2}L^{4}}},\;e_{w}=\left|\frac{w_{EB}-w_{IF}}{w_{IF}}\right|\;\approx \frac{1}{1+\frac{35E^{2}I^{2}}{3P^{2}L^{4}}}$$

which suggests that, within the scope of Euler‐Bernoulli beam theory, the deviation in the linear force‐displacement relationship is more pronounced than that in the force‐angle relationship since *e*_θ_ <  *e_w_* even when the applied force is small (Figure 1c). Consequently, the force‐angle relation is less sensitive to the neglected nonlinear term than the force‐displacement relation. This suggests that the angle can serve as a robust mechanical readout variable, especially when a simple analytical solution for force reconstruction is desired. Moreover, it is also assumed in small deformation that the arc coordinates (along the deformed beam) and the reference coordinate *x* are approximately equal to each other, something that will no longer be appropriate when beam deflection becomes large. Actually, it can be shown that, even under large deformation, the load *P* acting on a micropillar can be expressed in terms of its rotation angle *θ_L_* at the free end as:

(4) $$F\left(k\right)-F\;\left(k,\psi_{0}\right)=\sqrt{\frac{P}{EI}}\;L,\psi_{0}=\arcsin\frac{1}{\sqrt{2}k}\;,\;k=\sqrt{\frac{\sin\theta_{L}+1}{2}}\;$$

where *F*(*k*) and *F*(*k*, ψ_0_) respectively represent the complete and incomplete elliptic integral of the first kind (see Note S1 for details [19]). In practice, the pillar arrays made of PDMS are monolithic with the substrate. Therefore, the measured rotation angle of the pillar top *θ_M_* comprises contributions from bending of the beam *θ_L_* and tilting of the substrate *θ_T_*. We here introduced a factor (see Note S1 and Figure S2 for details):

(5) $$C_{\theta }=\frac{1}{\frac{4\left(1-\nu^{2}\right)}{3\pi }\frac{D}{L}+1}\;$$

to eliminate the effect of the tilting of the pillar base. The corrected angle *θ_L_* = *C_θ_ θ_M_* can be directly substituted into Equation (4) to quantify the lateral force applied on the pillar top. Using finite element simulations, we demonstrated that the newly proposed angle‐based force estimation method achieves exceptional accuracy, reducing the error of the displacement‐based method by up to ∼40% compared with the most basic analytical back‐calculation for micropillars with small aspect ratios (Figure 1d).

### 2.2 Integrating ODMR and LPM: Enabling 3D Orientation Tracking of FNDs

**FIGURE 2:**
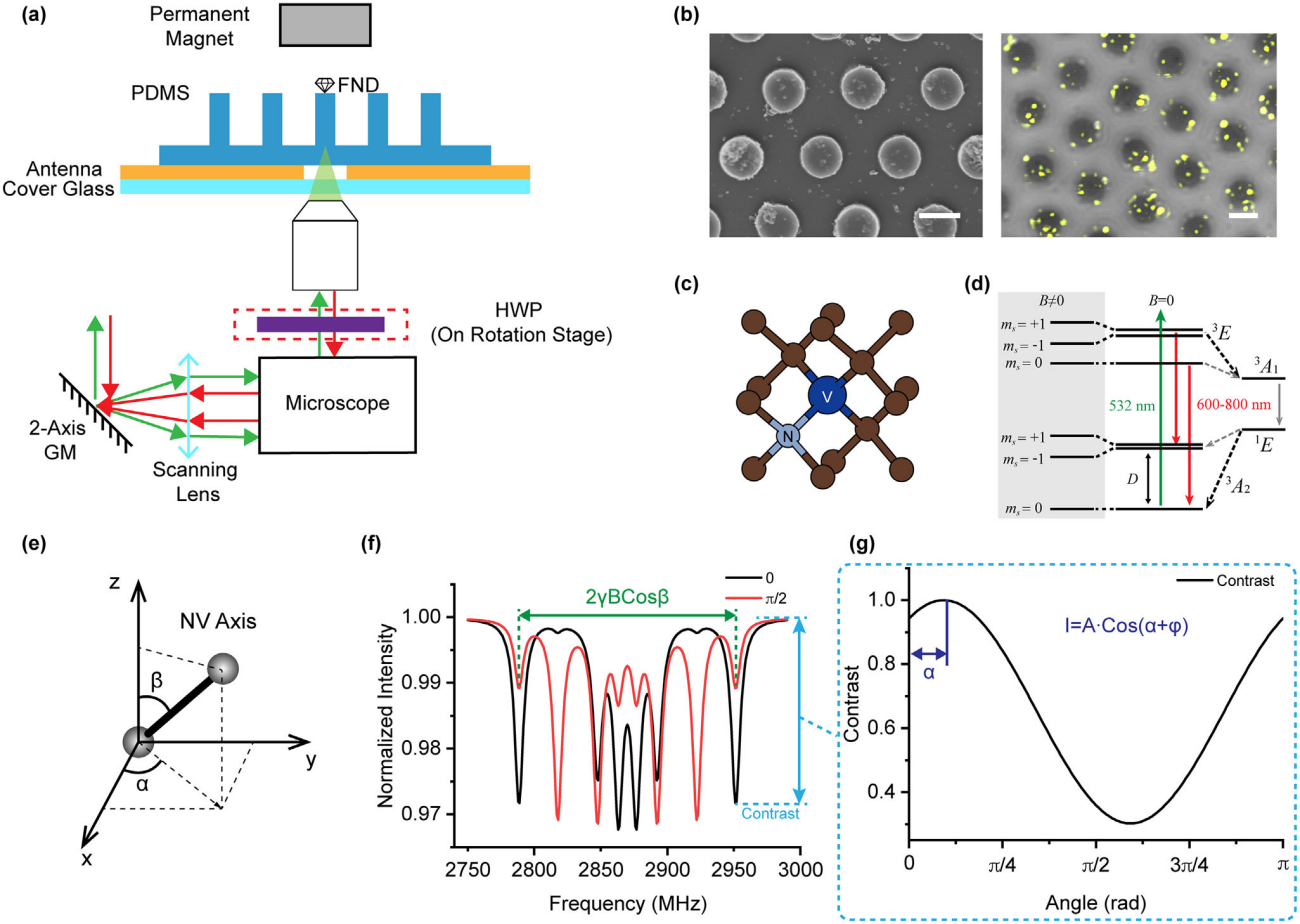
Quantum‐enabled 3D rotation measurement using the hybrid ODMR‐LPM method. (a) Optical path design diagram. (b) SEM image (left) and confocal image (right) of PDMS micropillar array with FNDs. The scale bar is 2 µm. (c) Illustration of NV center structure. (d) Energy level of the NV center. (e) Orientation of the NV axis and the corresponding angle. (f) Simulated ODMR spectrum for NV centers under different laser polarizations. (g) ODMR contrast response of laser polarization.

To translate the theoretical advantages of angle‐based force measurement into practice, we use FNDs containing NV centers as three‐dimensional rotation sensors. By combining the magnetic‐field‐dependent resonance of NV centers with their polarization‐dependent optical response, we integrate ODMR and LPM to overcome the limitations of each technique when used alone. As shown in Figure 2a, a home‐built confocal microscope system combining polarized optical excitation, microwave modulation, and an external magnetic field was developed for hybrid ODMR‐LPM angle measurements. FNDs were fixed at the tops of PDMS micropillars (Figure 2b), forming a stable nanodiamond coating with an average density of approximately six FNDs per pillar (Note S2, Figures S3–S6). These FNDs serve as orientation markers that convert pillar bending and rotation into measurable changes in FND crystal orientation.

The NV center is a point defect in diamond consisting of a substitutional nitrogen atom adjacent to a vacancy (Figure 2c). Its spin‐triplet ground state exhibits Zeeman splitting under an external magnetic field (Figure 2d), and the magnitude of this splitting depends on the angle between the NV axis and the magnetic‐field direction. This enables the determination of the relative orientation between each NV axis and the field. However, rotation around the magnetic‐field axis does not change this relative angle, making axial rotation inaccessible with a single magnetic field. Although multi‐field ODMR approaches can resolve this ambiguity, they significantly increase experimental complexity.

All‐optical polarization‐based methods, such as LPM, determine NV orientation through polarization‐dependent excitation or emission. It is sensitive to the in‐plane projection of the NV axis but cannot fully resolve the three‐dimensional orientation. Moreover, for FNDs containing ensembles of NV centers with multiple orientations, the superposition of polarization responses results in a single projected vector. According to Euler's rotation theorem, complete determination of rigid‐body rotation requires at least two non‐collinear vectors; therefore, LPM alone cannot reconstruct the full rotation matrix.

By integrating the ODMR and LPM techniques, the limitations inherent to each individual method can be overcome. The orientation‐dependent Zeeman splitting enables frequency‐domain separation of NV centers with different crystallographic axes, while LPM provides the rotation information around the magnetic‐field axis. To implement this strategy, a polarized laser, an external microwave, and a magnetic field are combined within a confocal microscope platform (detailed in Note S3 and Figure S7). A half‐wave plate mounted before the objective controls the excitation polarization. The microwave is fed to the sample through a co‐planar waveguide deposited on the cover glass. The magnetic field is generated by a permanent magnet, and its direction is carefully aligned to be vertical (the calibration is detailed in Note S4 and Figure S8). During the experiments, the permanent magnet needs to be moved away and returned to its original position. Therefore, we evaluated the magnet's relocation precision. The results show that it remained nearly unchanged (Figure S9). In this way, the NV center orientation can be separated into two independent angles (Figure 2e): a vertical angle β, extracted from the ODMR peak separation (Figure 2f), and a horizontal angle α, determined from the polarization‐dependent contrast variation of the corresponding ODMR peaks (Figure 2g; Figures S10 and S11; See Note S6 for the detailed calculation process). Because NV centers with different orientations are separated in the frequency domain, their polarization responses can be individually analyzed. With two or more non‐collinear NV axes, the rotational ambiguity associated with a single NV axis can be removed, allowing the full three‐dimensional orientation of the diamond particle to be determined.

Theoretically, the complete spatial orientation of the diamond particle can be formally described by three Euler angles (α, β and γ) following the ZXZ convention (detailed in SI Note 4). In our experimental setup, the angle α is determined using the LPM method with an accuracy of approximately 1°. Meanwhile, the angles β and γ are extracted from the ODMR measurements, achieving sensitivities of 0.87 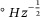 and 1.76 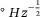, respectively (Figure S12).

We next verified its practical suitability for live‐cell applications. We investigated the stability of the measurement system under physiologically relevant conditions and confirmed that the observed variations were within the established measurement precision limits (Table S1). Furthermore, evaluating the cytotoxicity of the hybrid ODMR‐LPM protocol revealed no significant negative impact on cell viability (Figure S13). Together, these validations demonstrate that our technique delivers both the robust measurement stability and the biocompatibility required for continuous mechanobiological studies.

### 2.3 Validation of In‐Plane and Out‐of‐Plane Rotation Angle Measurement

**FIGURE 3:**
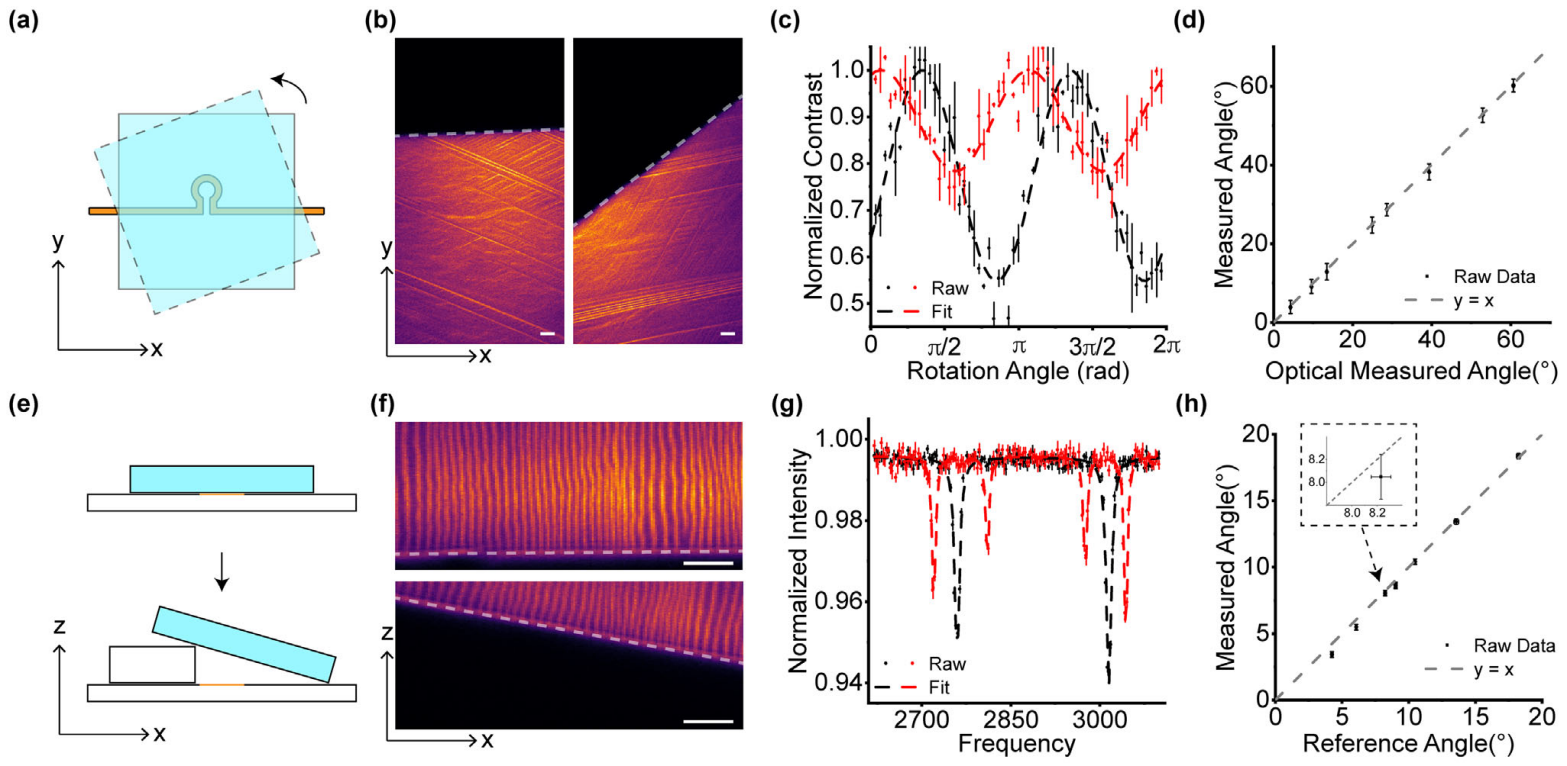
Experimental validation of the proposed hybrid ODMR‐LPM method for 3D rotation measurement. (a) Schematic of the in‐plane rotation of the bulk diamond. (b) Confocal image of the bulk diamond before (left) and after (right) the rotation. The edge of the diamond is labeled with a dashed line. (c) Typical measurement result of the LPM spectrum. The black points and line show the measured data and fitting result of the diamond before rotation. The red points and line show the measured data and fitting result of the diamond after rotation. (d) Comparison of the measured in‐plane rotation angle using the optical method and the hybrid ODMR‐LPM method. (e) Schematic of the out‐of‐plane rotation of the bulk diamond. (f) Confocal image of the bulk diamond before (left) and after (right) the rotation. The edge of the diamond is labeled with a dashed line. (g) Typical measurement result of the ODMR spectrum. The black points and line show the measured data and fitting result of the diamond before rotation. The red points and line show the measured data and fitting result of the diamond after rotation. (h) Comparison of the measured out‐of‐plane rotation angle using the optical method and the hybrid ODMR‐LPM method. The scale bars in (b) and (f) are 10 µm.

To validate the proposed rotation angle measurement method and evaluate its performance, we conducted both horizontal and vertical rotation experiments using a bulk diamond sample. As shown in Figure 3a, a [100]‐cut bulk diamond was placed on a cover glass coated with a coplanar waveguide. The diamond was randomly rotated in the horizontal plane, and at each rotation angle, both confocal scanning and hybrid ODMR‐LPM measurements were performed. The confocal image provides an optical reference for the hybrid ODMR‐LPM measurement. As shown in Figure 3b, due to the presence of color centers in the bulk diamond, a clear boundary corresponding to the edge of the diamond can be observed in the confocal image. The horizontal rotation angle was determined by comparing the boundary in images acquired at different rotation angles. During the hybrid ODMR‐LPM measurements, the ODMR spectrum showed almost no change, consistent with the method's mechanism: the relative angle between the NV centers and the applied magnetic field remains constant during in‐plane rotation. In contrast, the phase shift in the LPM spectrum clearly reveals the horizontal rotation, as shown in Figure 3c. By comparing the optical reference angle with the rotation angle extracted from the hybrid ODMR‐LPM method (Figure 3d), the horizontal rotation measurement demonstrates high accuracy.

The vertical rotation measurement method was validated similarly. As shown in Figure 3e, one edge of the bulk diamond remained in contact with the cover glass, while a thin quartz plate was inserted beneath the opposite side, causing the diamond to rotate by a certain angle in the vertical direction. The vertical rotation angle could be adjusted by varying the thickness of the inserted quartz plate. To determine the exact rotation angle, an XZ‐plane scan was performed with the confocal microscope, allowing visualization of the cross‐section of the bulk diamond, as shown in Figure 3f. The rotation angle obtained by comparing the boundaries in two images was treated as the optical reference, and the corresponding rotation angle was also extracted from the hybrid ODMR‐LPM measurement. Since the relative orientation between the NV centers and the magnetic field changes during out‐of‐plane rotation, a significant shift in the ODMR spectrum was observed (Figure 3g). By comparing the optical reference with the rotation angle measured by the hybrid ODMR‐LPM method (Figure 3h), the vertical rotation measurement shows high accuracy, with an error of approximately 0.5°, comparable to previously reported results from conventional ODMR methods.

### 2.4 3D Rotational Force Measurement in Cells

With the accuracy and precision of our 3D angular sensing platform validated, we next applied it to a relevant biological context: measuring the complex forces exerted by cells. We seeded NIH‐3T3 fibroblasts onto FND‐functionalized PDMS micropillar arrays. After 24‐h incubation, cells spread and generated contractile forces, inducing 3D pillar deformations. We subsequently fixed the cells, lysed them with proteinase K to release mechanical stress, and allowed the elastic pillars to return to their original positions. During this stress‐relaxation process, we simultaneously measured out‐of‐plane bending angles and in‐plane rotation angles of pillar tops using the hybrid ODMR‐LPM method, and recorded FND displacements via confocal imaging for conventional displacement‐based TFM analysis.

**FIGURE 4:**
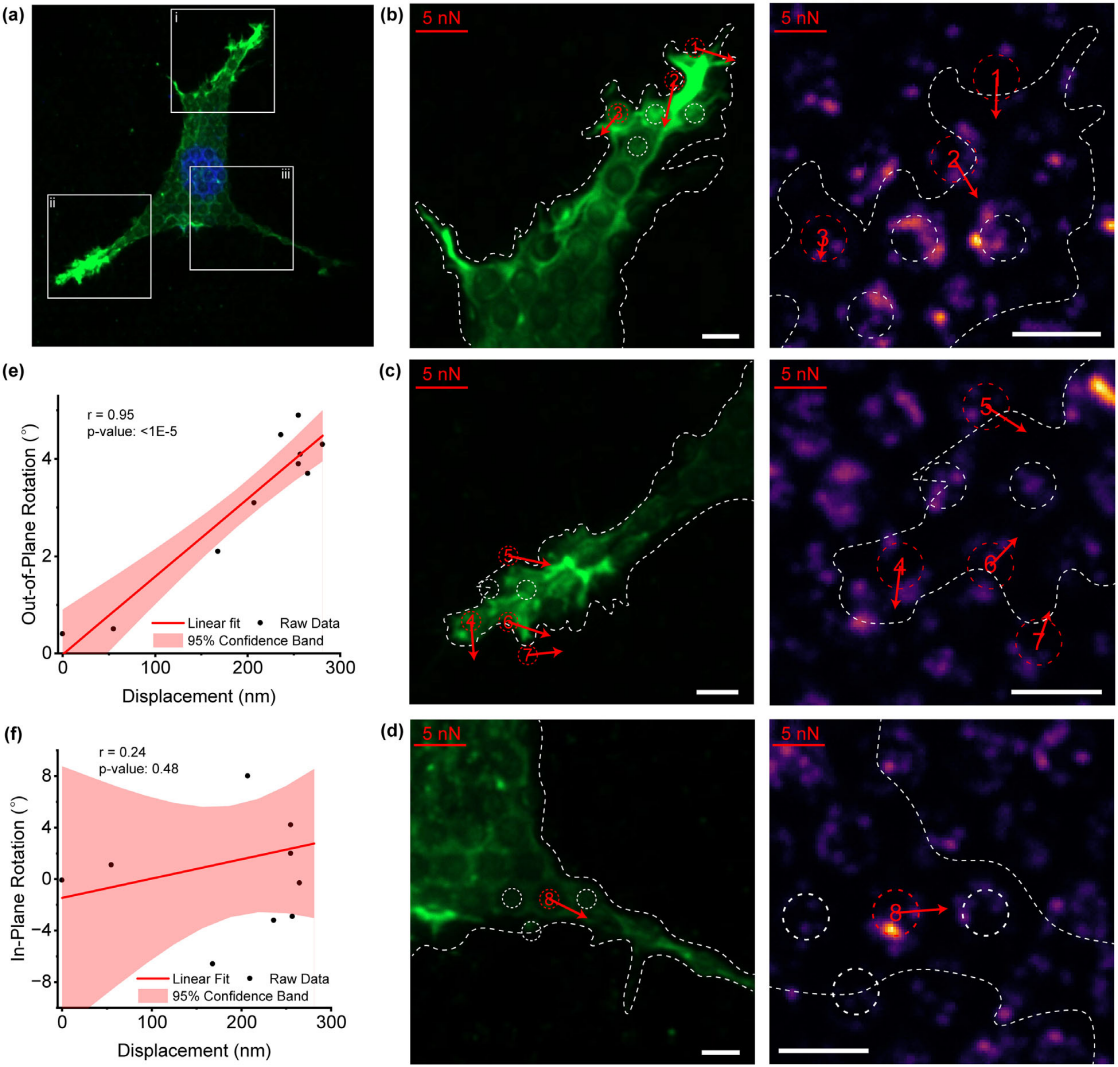
3D cellular force measurement using the hybrid ODMR‐LPM method. (a) Confocal image of the measured cell and three measured areas. (b–d) Cell force measurement using the displacement method (left panels) and the hybrid ODMR‐LPM method (right panels). The scale bars in all panels are 4 µm. (e) Correlation analysis of out‐of‐plane rotation and displacement. (f) Correlation analysis of in‐plane rotation and displacement.

We detected significant rotational signals at polarized regions of spread cells, including areas i, ii, and iii, shown in Figure 4a–d, where traction forces typically concentrate. Specifically, out‐of‐plane rotations ranged from 2.1^○^ to 4.9^○^, while in‐plane rotations ranged from 2^○^ to 13^○^. In contrast, non‐adhesion control regions exhibited out‐of‐plane rotations below 0.5^○^ and in‐plane rotations below 1.1^○^ (within measurement uncertainty), confirming the specificity of our mechanical readouts.

Correlation analysis revealed distinct relationships between rotational motions and pillar displacement (Figure 4e,f). Out‐of‐plane rotation exhibited a strong positive correlation with displacement, indicating that horizontal traction forces dominate pillar bending. Conversely, in‐plane rotation showed no significant correlation with displacement, suggesting that in‐plane rotations arise from more complex mechanical interactions. We hypothesize that in‐plane rotation originates from cellular traction but is strongly modulated by the eccentricity of force application. Theoretically, forces applied farther from the pillar's central axis generate a larger in‐plane rotation response. Consistently, pillars 1, 2, 5, and 7, where cellular adhesions localized near the upper edge of pillar tops, displayed more pronounced rotational motions due to their longer effective lever arms compared to pillars beneath the cell body.

More importantly, our 3D angular measurement system enables precise quantification of cellular traction forces on individual pillars. Angle‐derived forces were consistently lower than displacement‐based estimates (Table 1), a trend that aligns quantitatively with our theoretical predictions and finite‐element simulations. This systematic discrepancy arises because displacement methods, constrained by optical resolution limitations, overestimate actual pillar deformation under identical imaging conditions.

**TABLE 1:** Cell traction forces from angle and displacement methods. *The relative difference is calculated by the formula:$\;\frac{Force(Angle\;Method)-Force(Displacement\;Method)}{Force(Displacement\;Method)}$.

	In‐Plane Rotation	Out‐of‐Plane Rotation	Force (Angle Method)	Displacement	Force (Displacement Method)	Relative Difference*
Pillar 1	4.2°	3.9°	4.0 nN	255 nm	4.1 nN	−2.4%
Pillar 2	13.0°	4.3°	4.4 nN	281 nm	4.6 nN	−4.3%
Pillar 3	− 6.6°	2.1°	2.1 nN	168 nm	2.7 nN	−22%
Pillar 4	− 2.9°	4.1°	4.2 nN	257 nm	4.2 nN	−0%
Pillar 5	− 3.2°	4.5°	4.6 nN	236 nm	3.8 nN	+21%
Pillar 6	− 0.3°	3.7°	3.8 nN	265 nm	4.3 nN	−12%
Pillar 7	8.0°	3.1°	3.2 nN	207 nm	3.4 nN	−5.9%
Pillar 8	2.0°	4.9°	5.0 nN	255 nm	4.1 nN	+22%
Control 1	1.1°	0.5°		55 nm		
Control 2	− 0.1°	0.4°		0 nm		

In summary, our angular measurement platform successfully captures three‐dimensional mechanical signatures, out‐of‐plane rotation, and in‐plane rotation in cellular force fields and establishes a rotation‐based framework for accurate force quantification. This work provides a new paradigm for deciphering the complex mechanobiology of cell‐matrix interactions.

## 3 Discussion

The central advance of this work is a conceptual extension of pillar‐based traction force microscopy: from displacement‐only readouts to rotational‐angle‐based force measurement. Conventional micropillar and gel‐based traction force measurements infer cellular forces from lateral displacements using well‐established force‐deflection relationships. Our NV‐based approach extends the measurement capabilities of TFM by directly measuring pillar rotation via the orientation‐dependent optical response of NV centers. Rather than relying on displacement tracking, it uses angular determination as the primary readout for cellular force measurement. In our implementation, we achieve an angular precision of ∼0.5°, corresponding to a force precision equivalent to that produced by a ∼30 nm tip displacement in a conventional displacement‐based calibration with a 6 µm pillar (L/D = 3, 1.49 MPa). This precision is compatible with conventional deflection‐based TFM under similar conditions [30, 31], while providing rotational information not captured by conventional displacement‐based readouts.

From a mechanical standpoint, our analysis clarifies why angle can serve as a more robust variable at the same level of calculation convenience. Within the classical Euler–Bernoulli theory framework, neglecting the (*dw*/*dx*)^2^ term introduces a smaller relative error in the predicted rotation than in the predicted tip displacement, leading to systematically more accurate force estimates when angle is used as the calibration variable. Moreover, when large deformations occur, and the beam axis is significantly curved, the load–angle relationship remains expressible through elliptic integrals, while the displacement‐based linear calibration diverges. Finite‐element simulations confirm that our angle‐based method reduces force errors by at least 10% compared to the most basic traditional displacement‐based estimates with the linear Euler‐Bernoulli formula. Thus, within a simple analytical framework, angle‐based readout mitigates model errors associated with geometric nonlinearity and provides quantitatively reliable force estimation under realistic cellular loading conditions. We emphasize that this comparison is made against the simple analytical displacement‐based model rather than FEM‐based or nonlinear‐model‐based reconstruction, which can largely eliminate linear‐model error by back‐calculating traction forces from measured displacement data. In this context, the advantage of the angle‐based method lies in achieving high force accuracy without requiring additional computational reconstruction.

Beyond improving force accuracy, angle readout fundamentally enriches the type of mechanical information accessible from micropillar arrays. The 3D orientation of the pillar top encodes both out‑of‑plane bending and in‑plane rotation, allowing us to distinguish between centrally applied forces and off‐axis traction. In our cell experiments, we observed in‐plane rotations that did not correlate with tip displacement, particularly on pillars where focal adhesions were located near the edge of the pillar top. This observation is consistent with a simple lever‐arm picture: forces applied farther from the pillar axis produce larger in‐plane rotations for the same net traction. Such in‐plane rotational signals are largely invisible to conventional displacement‐only readouts. Yet, they highlight that conventional displacement‐only readouts discard the rotational component of cellular force transmission, thereby missing a potentially important dimension of cell‐matrix mechanical interactions. In future work, systematic mapping of bending and in‐plane rotation across different cell types and perturbations could reveal whether rotational components of cellular forces play a role in mechano‐transduction—for example, in directional migration, matrix remodeling, or durotaxis.

At the same time, the present implementation of the hybrid ODMR‐LPM platform has several technical limitations that motivate further development. First, the external magnetic field is provided by a fixed permanent magnet, which ensures excellent stability but limits field uniformity and alignment flexibility across the field of view. Spatial variations in field magnitude and direction can introduce systematic errors in the extracted NV orientation. Replacing the permanent magnet with a well‑characterized electromagnet or Helmholtz‑coil configuration would improve field homogeneity and allow dynamic control of the field direction, increasing robustness and enabling more flexible measurement protocols. A second limitation is measurement throughput. In the current proof‐of‐concept implementation, the hybrid ODMR‐LPM readout is performed through confocal microscopy with sequential ODMR spectrum and polarization scans. In addition, random FND distribution can result in multiple FNDs with different crystallographic orientations on a single micropillar, leading to signal overlap and requiring confocal isolation of individual emitters. This makes the current implementation a point‐by‐point readout, limiting its suitability for high‐speed, continuous live‐cell traction mapping. Future development should therefore focus on enabling parallel readout and controlling FND placement. On the optical side, super‐resolution‐enabled wide‐field ODMR imaging [32], together with rapid microwave and polarization modulation, could enable parallel readout of pillar rotations. On the sample‐preparation side, patterned placement of single nanodiamonds [33] would reduce signal overlap and minimize the need for confocal isolation. Together, these improvements provide a path to transforming the current platform into a higher‐throughput implementation capable of tracking dynamic cellular forces in living systems.

Taken together, our results establish rotational angle as a rigorous and experimentally accessible mechanical observable for micropillar‑based traction force measurements and demonstrate that diamond‑based 3D orientation sensing provides a practical route to implement this concept. By integrating FNDs with PDMS micropillar arrays, we transform a conventional displacement sensor into a quantum‑enhanced, rotation‑angle‑based cellular force probe capable of resolving both translational forces and rotational deformations at the single‑pillar level. This rotation‑based framework extends the capabilities of micropillar platforms from scalar force readout toward high‑precision, 3D biomechanical sensing. With further technical refinement, especially in magnetic‑field control and parallel, high‑speed readout, angular traction microscopy offers a promising pathway for investigating complex, time‑dependent mechanobiological processes and microscale material responses.

## 4 Conclusion

In conclusion, we introduce an angle‐based framework for quantifying cell traction forces using PDMS micropillar arrays. Theoretical analysis shows that with a simple analytical framework, rotational angle provides a more robust force estimation than conventional linear displacement‐based estimation, particularly when geometric nonlinearity becomes non‐negligible. This provides a solid mechanical foundation for using the rotation angle as an alternative readout for force reconstruction in pillar‐based TFM. By integrating diamond‐based orientation sensing with a hybrid ODMR‐LPM measurement method, we directly measure the rotational angle at the pillar top. This approach transforms the conventional PDMS micropillar array into a quantum‐enhanced rotation‐angle‐based cellular force sensor. Moreover, the proposed framework extends the capabilities of micropillar platforms toward high‐precision, 3D biomechanical sensing. With further technical refinement, it provides a promising pathway for investigating complex biological and material systems at the microscale.

## 5 Methods

### 5.1 Sample Preparation

An omega‐shaped coplanar microwave antenna was fabricated directly on a glass coverslip by thermal evaporation of a Ti/Au/Ti (titanium/gold/titanium) tri‐layer [34]. The PDMS micropillar array was fabricated using standard soft lithography techniques. Each pillar measured 6 µm in height and 2 µm in diameter. PDMS (Sylgard 184, Dow Corning) was mixed with its curing agent at a 10:1 ratio and poured onto 1 cm × 1 cm silicon molds. The mixture was degassed under vacuum for 30 min and subsequently covered with the glass coverslip containing a coplanar microwave antenna. The samples were cured in an oven at 80 ^○^ *C* overnight and subsequently peeled from the silicon molds. The fabricated pillar arrays were cylindrical, with a center‐to‐center spacing equal to twice the pillar diameter, thereby maintaining a uniform area density.

Measurement of the Young's modulus is recommended for each batch, as the mechanical properties of PDMS may vary with the synthesis conditions, while the interpreted cellular forces are directly determined by it. The average Young's modulus of the synthesized samples was 1.4 ± 0.5 MPa.

To ensure that the PDMS layer on the coverslip remained thinner than 50 µm, the amount of PDMS dispensed onto the silicon molds was controlled within 5–10 mg. This limitation was required to ensure that the total thickness did not exceed the working distance of the microscope objective.

### 5.2 FND Coating

The surface of the PDMS micropillar array was functionalized by immersion in 3‐aminopropyl triethoxysilane (APTES) solution in ethanol (8%, v/v) for 10 min, followed by sequential rinsing with ethanol (99.8%) and deionized (DI) water. FNDs (0.025 mg/mL in DI water) were dispersed using an ultrasonic water bath. The PDMS micropillar array was incubated with the dispersed FND solution for 10 min, after which unbound FNDs were removed by rinsing with DI water.

### 5.3 Confocal Measurement

The confocal images in this study were acquired using two different setups. To locate FNDs for ODMR and LPM experiments, we used a custom‐built confocal microscope (as described in the Supplementary Information) equipped with an air objective (UPLANXAPO40X, Olympus) with a numerical aperture of 0.95 NA, and a 532 nm continuous‐wave green laser provided excitation at a power of 5 µW.

For illustrative purposes, including high‐resolution imaging of the cell and sample structure and three‐dimensional optical sectioning, we used a commercial confocal microscope (LSM 980, Zeiss).

### 5.4 ODMR Measurement

The microwave required for the ODMR experiments was generated by a microwave source (SynthNV Pro, Windfreak Technologies), amplified using a high‐power amplifier (ZHL‐16W‐43‐S+, Mini‐Circuits), and delivered to the sample through an omega‐shaped coplanar waveguide patterned on the glass coverslip. The reference channel monitored baseline fluorescence for normalization, while the signal channel captured the microwave‐modulated fluorescence response of the NV centers. The total integration time for each frequency point is 54 ms, and each loop is repeated 10 times.

### 5.5 LPM Measurement

In LPM measurements, a mechanical rotation stage rotated a half‐wave plate (HWP), thereby modulating the polarization of the excitation laser. During the experiment, the rotation speed was set to 15 degrees per second. While the stage rotated, the previously described microwave sequence was continuously applied to the sample, and both the reference fluorescence intensity and the signal intensity were simultaneously recorded in two separate photon‐counting channels. The rotation stage completed a full 360° cycle, and fluorescence intensity data were recorded at 3° intervals.

### 5.6 Cell Culture

NIH‐3T3 cells were cultured in T‐25 flasks. The complete growth medium consisted of DMEM (Gibco) supplemented with 10% (v/v) fetal bovine serum (FBS; Invitrogen), 2 mm L‐glutamine (Invitrogen), and 100 U/mL penicillin‐streptomycin (Invitrogen). Cells were maintained at 37°C in a humidified incubator with 5% CO_2_.

Before cell seeding, the FND sensor array was coated with human plasma fibronectin solution (50 µg/mL; Thermo) and incubated at 4°C overnight. The array was then rinsed three times with phosphate‐buffered saline (PBS) and sterilized under UV light for 20 min. Cells were trypsinized, resuspended in DMEM, and seeded onto the sensor array for 24 h.

### 5.7 Fluorescence Staining

Cells cultured on the sensor array were fixed with 4% paraformaldehyde for 10 min at room temperature. Following fixation, cells were permeabilized with 0.25% Triton X‐100 in PBS for 5 min at room temperature.

Nuclear staining was performed using Hoechst 33342 (Invitrogen; 1:700 dilution), and filamentous actin was labeled with Phalloidin (Abcam, iFluor 488, ab176753; 1:1000 dilution). Staining was carried out for 1 h at room temperature. Finally, cells were rinsed three times with PBS and imaged using a Zeiss LSM 980 confocal microscope.

## Author Contributions

**Yong Hou**: project administration, supervision, Writing – review and editing, Writing – original draft, formal analysis, conceptualization, methodology. **Linjie Ma**: investigation, writing – original draft, formal analysis, methodology, data curation, validation, visualization, writing – review and editing. **Tai Nam Yip**: methodology, investigation, validation, writing – review and editing. **Yuan Lin**: writing – review and editing, project administration, supervision, resources, funding acquisition, conceptualization. **Bicong Wang**: writing – original draft, formal analysis, methodology, investigation, data curation, validation, software, visualization, writing – review and editing. **Yicheng Wang**: resources. **Zhiqin Chu**: writing – review and editing, project administration, supervision, resources, funding acquisition, conceptualization. **Jiahua Zhang**: resources. **Luyao Zhang**: resources.

## Conflicts of Interest

The authors declare no conflicts of interest.

## Supporting information




**Supporting File**: advs76685‐sup‐0001‐SuppMat.docx.

## Data Availability

The data that support the findings of this study are available from the corresponding author upon reasonable request.
