# Supplementary material for: From Displacement to Angle: Diamond‐Based 3D Rotation Sensing for High‐Precision Cellular Force Measurement
Source: Adv Sci (Weinh). 2026 Jul 20:e76685. Online ahead of print. doi: 10.1002/advs.76685 (PMC13383150; doi:10.1002/advs.76685)
Supplement: Supplementary file 1 — Supporting File: advs76685‐sup‐0001‐SuppMat.docx. [file ADVS-9999-e76685-s001.docx]

Supporting Information

From Displacement to Angle: Diamond-Based 3D Rotation Sensing for High-Precision Cellular Force Measurement

Linjie Ma^1#^, Bicong Wang^2#^, Tai Nam Yip^1^, Jiahua Zhang^1^, Yicheng Wang^1^, Luyao Zhang^1^, Yong Hou^1^*, Yuan Lin^2^*, and Zhiqin Chu^1^*

1. Department of Electrical and Computer Engineering, The University of Hong Kong, Hong Kong SAR

2. Department of Mechanical Engineering, The University of Hong Kong, Hong Kong SAR

*Corresponding authors:

Dr. Yong Hou, Email: houyong@eee.hku.hk

Prof. Dr. Yuan Lin, Email: ylin@hku.hk

Prof. Dr. Zhiqin Chu, E-mail: zqchu@eee.hku.hk

^#^ These authors made equal contributions to this work.

**Supporting Information Note 1: Model of bending pillar**

**Deformation of the cantilever beam**

When a cantilever beam is applied a concentrated force *P* at the free end, as described by **Figure S1**, its curvature is given by the equation:

$\frac{1}{\rho}=\frac{d\theta}{ds}$ (S1)

where *ρ* is the radius of curvature of the neutral surface. Additionally, if the beam in pure bending is linearly elastic and follows Hooke's law, the curvature is:

$\frac{1}{\rho}=\frac{M\left( s \right)}{EI}=\frac{P\left[ x\left( L \right)-x\left( s \right) \right]}{EI}$.

The sign conventions to be used with **Equation S2** are as follows: (1) the *x*-axis is positive to the right; (2) the rotational angle *θ* is positive when clockwise from the *x*-axis; (3) the deflection *w* is positive downward; (4) the bending moment is positive when it produces stretching in the upper part of the beam.


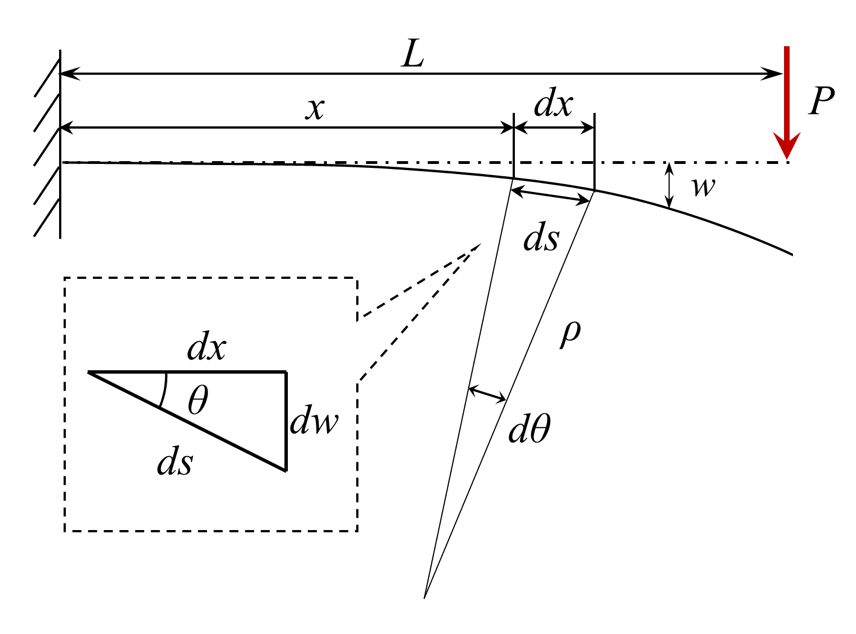


**Figure S1.** Bending of a beam.

The classical Euler-Bernoulli beam theory assumed that the deformations are very small, and the plane sections remain plane. Combing **Equation S1** and **Equation S2**, we can get:

$\frac{d\theta}{ds}=\frac{d\theta}{dx}\frac{dx}{ds}=\frac{d\theta}{dx}\frac{1}{\sqrt{1+\left( \frac{dw}{dx} \right)^{2}}}=\frac{P\left( L-x \right)}{EI}$. (S3)

From the inset of Figure S1, we see that:

$\theta=\arctan\frac{dw}{dx},\text{ }\frac{d\theta}{dx}=\frac{d^{2}w}{dx^{2}}\frac{1}{1+\left( \frac{dw}{dx} \right)^{2}}$,

With the assumption of small deflections, 1+(*dw*/*dx*)^2^ ≈ 1, we obtain^[1]^:

$\frac{d^{2}w_{EB}}{dx^{2}}\approx\frac{d\theta_{EB}}{dx}\approx\frac{P\left( L-x \right)}{EI}$.

Thus, the rotational angle *θ_EB_*(*L*) = *PL*^2^/(2*EI*) and deflection *w_EB_*(*L*) = *PL*^3^/(3*EI*).

Here, to estimate the errors brought by 1+(*dw*/*dx*)^2^ ≈ 1, we rewrite **Equation S3** by substituting *dw*/*dx* = tan *θ*:

$\left| \cos\theta\right|d\theta=\frac{P}{EI}\left( L-x \right)dx$.

Typically, the maximum angle of rotation at *x* = *L* is smaller than π/2, resulting |cos *θ*(*x*)| = cos *θ*(*x*). Considering the boundary condition *θ* (0) = 0, we can obtain:

$\theta_{IF}=\arcsin\left[ \frac{P}{EI}\left( Lx-\frac{x^{2}}{2} \right) \right]=\sum_{n=0}^{\infty} \frac{(2n)!}{4^{n}\left( n! \right)^{2}\left( 2n+1 \right)}\frac{P^{2n+1}}{E^{2n+1}I^{2n+1}}\left( Lx-\frac{x^{2}}{2} \right)^{2n+1}$.

It is noted that the expression of **Equation S7** reduces to the classical Euler–Bernoulli solutions when the terms with *n* > 0 are ignored. The deflection curve:

$w_{IF}=\int_{0}^{x} \tan\theta_{IF}dx=\int_{0}^{x} \frac{\sin\theta_{IF}}{\sqrt{1-{sin}^{2} \theta_{IF}}}dx$.

The integral of the above equation yields an incomplete elliptic integral, which cannot be expressed in terms of elementary functions. Thus, we use the series expansion for the tangent function:

$$w_{IF}=\int_{0}^{x} \tan\theta_{IF}dx=\int_{0}^{x} \left( \theta_{IF}+\frac{1}{3}\theta_{IF}^{3}+\cdots\right)dx$$

$$=\int_{0}^{x} \left[ \left( \frac{P}{EI}\left( Lx-\frac{x^{2}}{2} \right)+\frac{1}{6}\frac{P^{3}}{E^{3}I^{3}}\left( Lx-\frac{x^{2}}{2} \right)^{3}+\cdots\right)+\frac{1}{3}\left( \frac{P}{EI}\left( Lx-\frac{x^{2}}{2} \right)+\cdots\right)^{3}+\cdots\right]dx$$

$=\frac{P}{EI}\left( \frac{Lx^{2}}{2}-\frac{x^{3}}{6} \right)+\frac{P^{3}}{2E^{3}I^{3}}\left( \frac{1}{4}L^{3}x^{4}-\frac{3}{10}L^{2}x^{5}+\frac{3}{24}Lx^{6}-\frac{x^{7}}{56} \right)+\cdots$.

Through the above derivation, it can be shown that approximating 1+(*dw*/*dx*)^2^ by 1 gives rise to errors in rotational angle and deflection at *x* = *L* of:

$$e_{\theta}=\left| \frac{\theta_{EB}-\theta_{IF}}{\theta_{IF}} \right|\approx\frac{1}{1+\frac{\left( 24E^{2}I^{2} \right)}{\left( P^{2}L^{4} \right)}}$$

$e_{w}=\left| \frac{w_{EB}-w_{IF}}{w_{IF}} \right|\approx\frac{1}{1+\frac{\left( 35E^{2}I^{2} \right)}{\left( 3P^{2}L^{4} \right)}}$

It suggests that, within the framework of Euler-Bernoulli beam theory (Equation S3), the deviation in the linear force-displacement relationship is more pronounced than that in the force-angle relationship since $e_{\theta}< e_{w}$ even when the applied force is small (**Figure 1c**)

However, even **Equation S7** and **S8** compensate for the error arising from the approximation 1+(*dw*/*dx*)^2^ ≈ 1, they remain relatively high accuracy only under small deformations. This is due to a more subtle approximation, *x*(*s*) ≈ *s*, introduced during the derivation of Equation S3. Clearly, this approximation loses its reliability under large deformations. We can return to Equation S1 and S2 and re-derive the relationship between load and deformation. By taking the derivative with respect to *s*, we obtain a second-order ordinary differential equation as follows:

$\frac{d^{2}\theta}{ds^{2}}=-\frac{P}{EI}\frac{dx}{ds}=-\frac{P}{EI}\cos\theta$,

which can be solved as:

$\frac{1}{2}\left( \frac{d\theta}{ds} \right)^{2}=\frac{P}{EI}\left( \sin\theta_{L}-\sin\theta\right)$

with *θ_L_* being the realistic rotational angle of the free end of the beam. The above expression implies the implicit relationship between *θ* and *P*:

$\int_{0}^{\theta_{L}} \frac{d\theta}{\sqrt{\sin\theta_{L}-\sin\theta}}=\int_{0}^{L} \sqrt{\frac{2P}{EI}}ds$.

By introducing substitution sin *θ* = 2*k*^2^sin^2^*ψ*-1 and 2*k*^2^ = sin *θ_L_* +1, Eq. S11 can be derived into:

$\int_{\arcsin\frac{1}{\sqrt{2}k}}^{\frac{\pi}{2}} \frac{1}{\sqrt{2}k\sqrt{1-{sin}^{2} \psi}}\frac{2k\cos\psi d\psi}{\sqrt{1-k^{2}{sin}^{2} \psi}}=\int_{\arcsin\frac{1}{\sqrt{2}k}}^{\frac{\pi}{2}} \frac{\sqrt{2}d\psi}{\sqrt{1-k^{2}{sin}^{2} \psi}}=\sqrt{\frac{2P}{EI}}L$.

which is usually expressed in terms of elliptic integrals of the first kind^[2]^:

$F\left( k \right)-F\left( k,\psi_{0} \right)=\sqrt{\frac{P}{EI}}L,\psi_{0}=\arcsin\frac{1}{\sqrt{2}k}, k=\sqrt{\frac{\sin\theta_{L}+1}{2}}$.

where $F\left( k \right)$ and $F\left( k,\psi_{0} \right)$ respectively represent the complete and incomplete elliptic integral of the first kind.

**Tilting of the pillar base**

The preceding section derived the deformation of a beam with a fixed end at *x* = 0. However, the pillar arrays made of polydimethylsiloxane (PDMS) are monolithic with the substrate. When a lateral force is applied to the top of the pillar, the pillar base, where they connect to the substrate, deforms under the action of the torque *PL* (**Figure S2a**) ^[3-4]^. The tilting angle of the pillar base can be directly calculated by arctan(*u_A_*/*R*). The stress distribution at the bottom cross-section is given as follows:

$\sigma\left( y,z \right)=-\frac{PLy}{I},\text{ }y^{2}+z^{2}\leq R^{2}$.

For a soft elastic half-space, the vertical displacement caused by a concentrated force q is given by the Boussinesq solution^[5]^:

$u=\frac{1-\nu^{2}}{\pi E}\frac{q}{r}$,

where *r* is the distance from the concentrated force, *ν* is the Poisson ratio. Thus, the vertical displacement at *B* (*y_B_*, *z_B_*) (**Figure S2b**, *y_B_* ≤ 0) due to *σ* (*y*, *z*) with area *dydz*:

$u\left( y,z \right)=-\frac{1-\nu^{2}}{\pi E}\frac{PLy}{I}\frac{dydz}{\sqrt{\left( y-y_{B} \right)^{2}+\left( z-z_{B} \right)^{2}}}$.

Here, we only consider *z_B_* = 0 for simplicity. Introducing substitution *y* = *y_B_* + *r* cos *φ*, *z* = *r* sin *φ*:

$u\left( r,\varphi\right)=-\frac{1-\nu^{2}}{\pi E}\frac{PL}{I}\left( y_{B}+r\cos\varphi\right)drd\varphi$.

Then the total displacement at *B* is:

$u_{B}=-\frac{1-\nu^{2}}{\pi E}\frac{PL}{I}\int_{0}^{\pi} \int_{r_{1}}^{r_{2}} \left( y_{B}+r\cos\varphi\right)drd\varphi$,

where $r_{1,2}=\left( -y_{B}\cos\varphi\right)\mp\sqrt{R^{2}-y_{B}^{2}{sin}^{2} \varphi}$, thus,

$u_{B}=-\frac{1-\nu^{2}}{\pi E}\frac{PL}{I}\int_{0}^{\pi} -2y_{B}{sin}^{2} \varphi\sqrt{R^{2}-y_{B}^{2}{sin}^{2} \varphi}d\varphi$.

Introducing substitution *h* = -*y_B_* sin φ,

$$u_{B}=-\frac{1-\nu^{2}}{\pi E}\frac{PL}{I}\cdot2\int_{0}^{-y_{B}} \frac{2y_{B}\sqrt{R^{2}-h^{2}}}{\sqrt{y_{B}^{2}-h^{2}}}dh=-\frac{1-\nu^{2}}{\pi E}\frac{PL}{I}\frac{4}{y_{B}}\int_{0}^{-y_{B}} \frac{h^{2}\sqrt{R^{2}-h^{2}}}{\sqrt{y_{B}^{2}-h^{2}}}dh$$

$$\text{ }=-\frac{1-\nu^{2}}{\pi E}\frac{PL}{I}\frac{4}{y_{B}}\left[ \int_{0}^{-y_{B}} \frac{R^{2}h^{2}}{\sqrt{R^{2}-h^{2}}\sqrt{y_{B}^{2}-h^{2}}}dh-\int_{0}^{-y_{B}} \frac{h^{4}}{\sqrt{R^{2}-h^{2}}\sqrt{y_{B}^{2}-h^{2}}}dh \right]$$

$\text{ }=-\frac{1-\nu^{2}}{\pi E}\frac{PL}{I}\frac{4}{3}\left[ \left( \frac{R^{3}}{y_{B}}-Ry_{B} \right)F\left( \frac{\left| y_{B} \right|}{R} \right)+\left( 2Ry_{B}-\frac{R^{3}}{y_{B}} \right)E\left( \frac{\left| y_{B} \right|}{R} \right) \right]$,

where *F*(|*y_B_*|/*R*) and *E* (|*y_B_*|/*R*) are respectively the first kind and second kind complete elliptic integrals with modulus (|*y_B_*|/*R*). The vertical displacement at the edge point *A* is:

$u_{A}=\frac{4\left( 1-\nu^{2} \right)PLR^{2}}{3\pi EI}E\left( 1 \right)=\frac{4\left( 1-\nu^{2} \right)PLR^{2}}{3\pi EI}$.

Hence, the tilting angle of the pillar base *θ_T_* can be calculated by:

$\theta_{T}=\arctan\frac{u_{A}}{R}\approx\frac{4\left( 1-\nu^{2} \right)PLR}{3\pi EI}$.


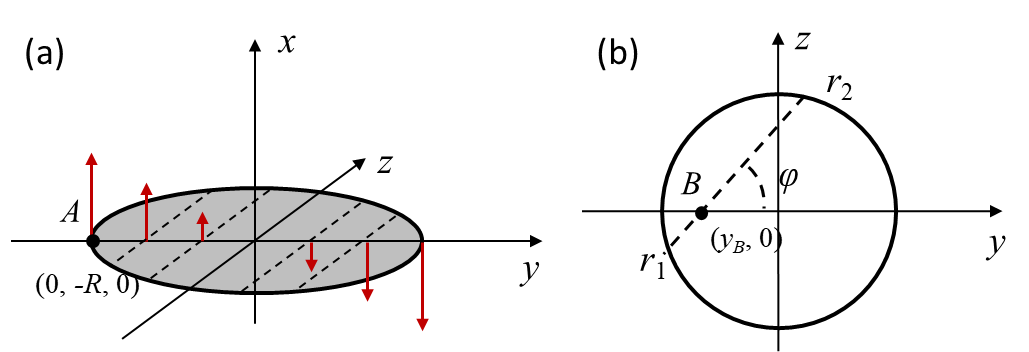


**Figure S2.** a) Stress distribution at the pillar bottom. b) Displacement at an internal point *B*.

In practice, the rotation angle of the pillar top measured by fluorescent nanodiamond (FND) *θ_M_* comprises contributions from bending of the beam *θ_L_* and tilting of the base *θ_T_*. We can estimate the proportion of *θ_L_* relative to *θ_M_* through the Euler–Bernoulli beam theory:

$C_{\theta}=\frac{\theta_{L}}{\theta_{M}}=\frac{\frac{PL^{2}}{2EI}}{\frac{PL^{2}}{2EI}+\frac{4\left( 1-\nu^{2} \right)PLR}{3\pi EI}}=\frac{1}{\frac{4\left( 1-\nu^{2} \right)}{3\pi}\frac{D}{L}+1}$.

That is, the measured angle *θ_M_* will be first multiplied by a correction factor *C_θ_* before being applied to **Equation S12** for force calculation.

**Numerical simulation**

We conducted finite element simulations with circular pillar height of 6 μm, Poisson ratio 0.47 and Young’s Modulus 1.49 MPa. Diameters were set as 1.5, 2, and 3 μm, respectively. The substrate measured 10^3^ μm^3^ with fixed boundaries at its bottom and surrounding. The traction force was respectively applied as a homogenous facial load *p* to the top surface of the pillar. The mesh was extra refined, and geometric nonlinearity was included. The rotational angle of the top surfaces was calculated from the vertical displacements of edge points.

**Supporting Information Note 2: Characterization of samples**

**FND coating on the PDMS micropillar**


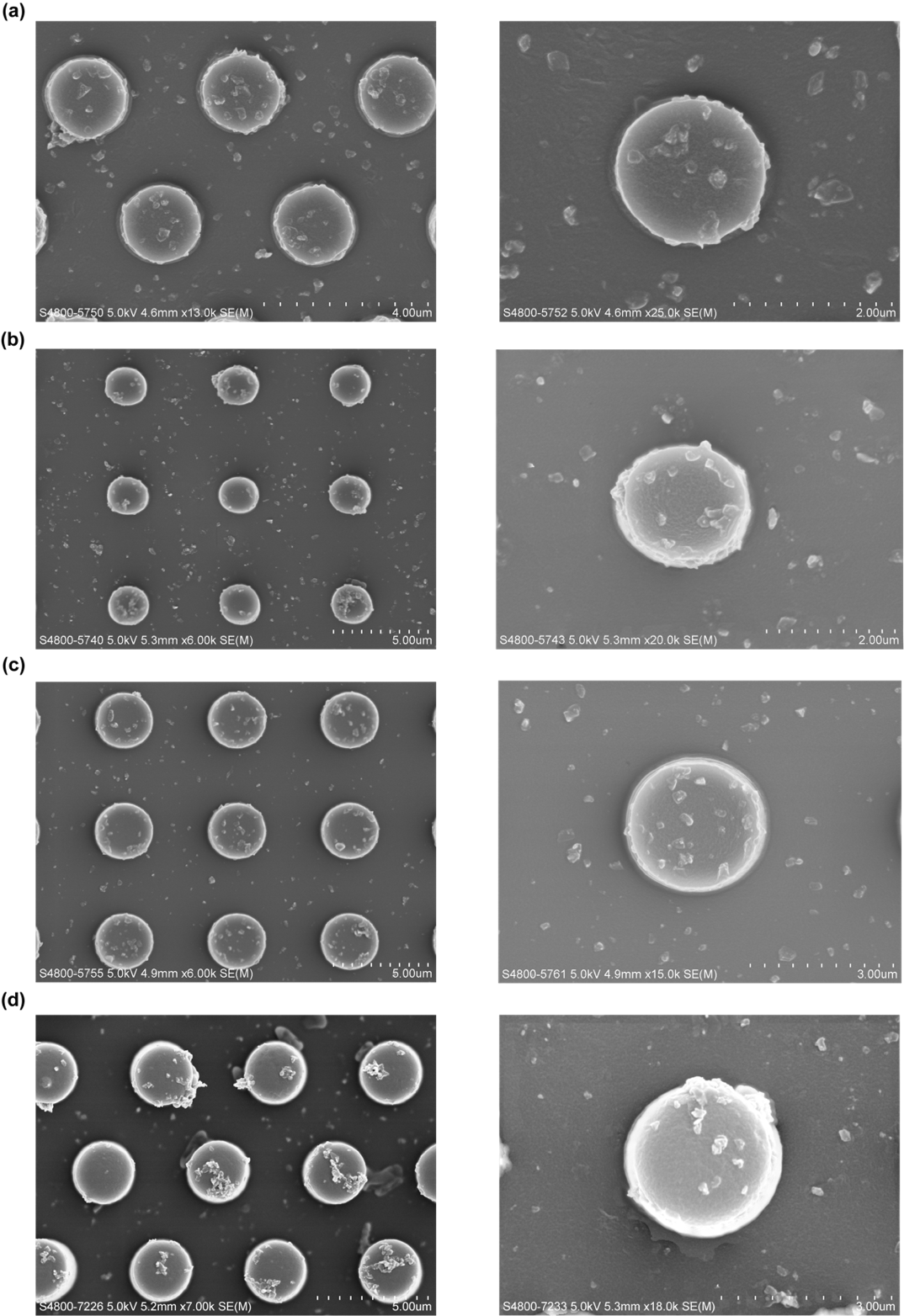


**Figure S3.** SEM images of FND-coated PDMS micropillar arrays with various geometries used in the experiments. All pillars have a height of 6 μm. a) 2 μm diameter, 4 μm center-to-center spacing. b) 2 μm diameter, 6 μm center-to-center spacing. c) 3 μm diameter, 6 μm center-to-center spacing. d) 2.5 μm diameter, 5.5 μm center-to-center spacing. Single-pillar SEM images represent depth-of-field–extended composites formed by merging images acquired at different focal planes (pillar top and substrate) due to insufficient depth of field.


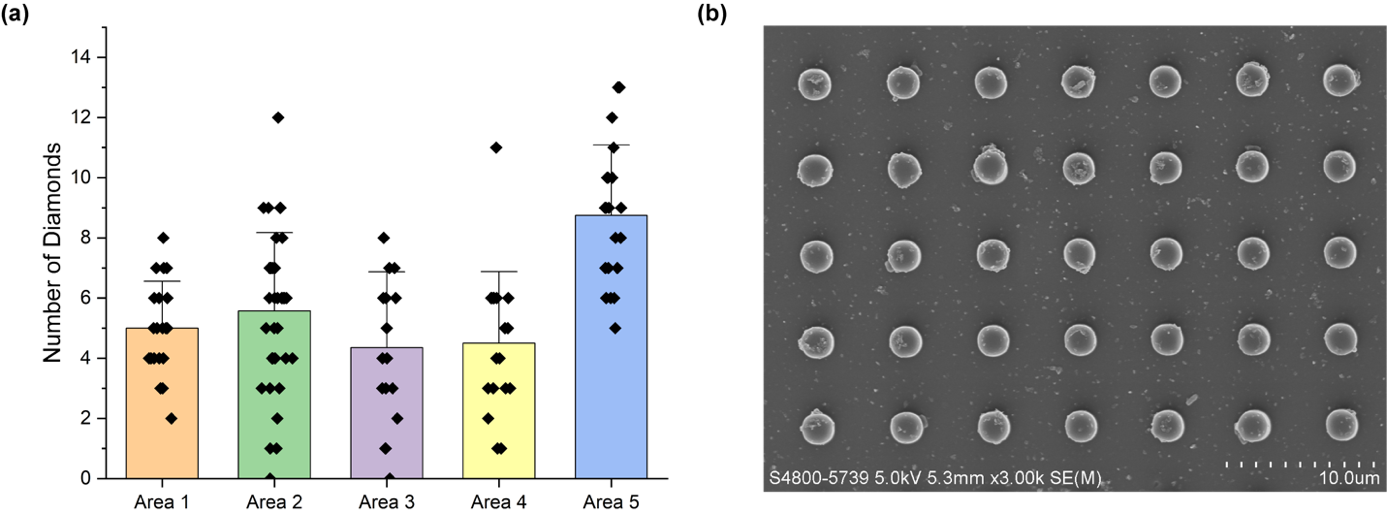


**Figure S4.** The statistical result of the FND coating. a) Statistics of the number of FNDs on the pillar top for 5 different areas. b) SEM image of a large area.

The statistical results shown in **Figure S4(a)** were obtained from SEM images of five randomly selected areas. A total of 110 PDMS micropillars were analyzed to determine the number of FNDs per pillar. The average number of FNDs per pillar is 5.7, with a standard deviation of 2.8. **Figure S4(b)** presents an SEM image over a larger area, demonstrating that nearly all pillar tops contain at least 2–3 FNDs.

**Stability of the FND coating**


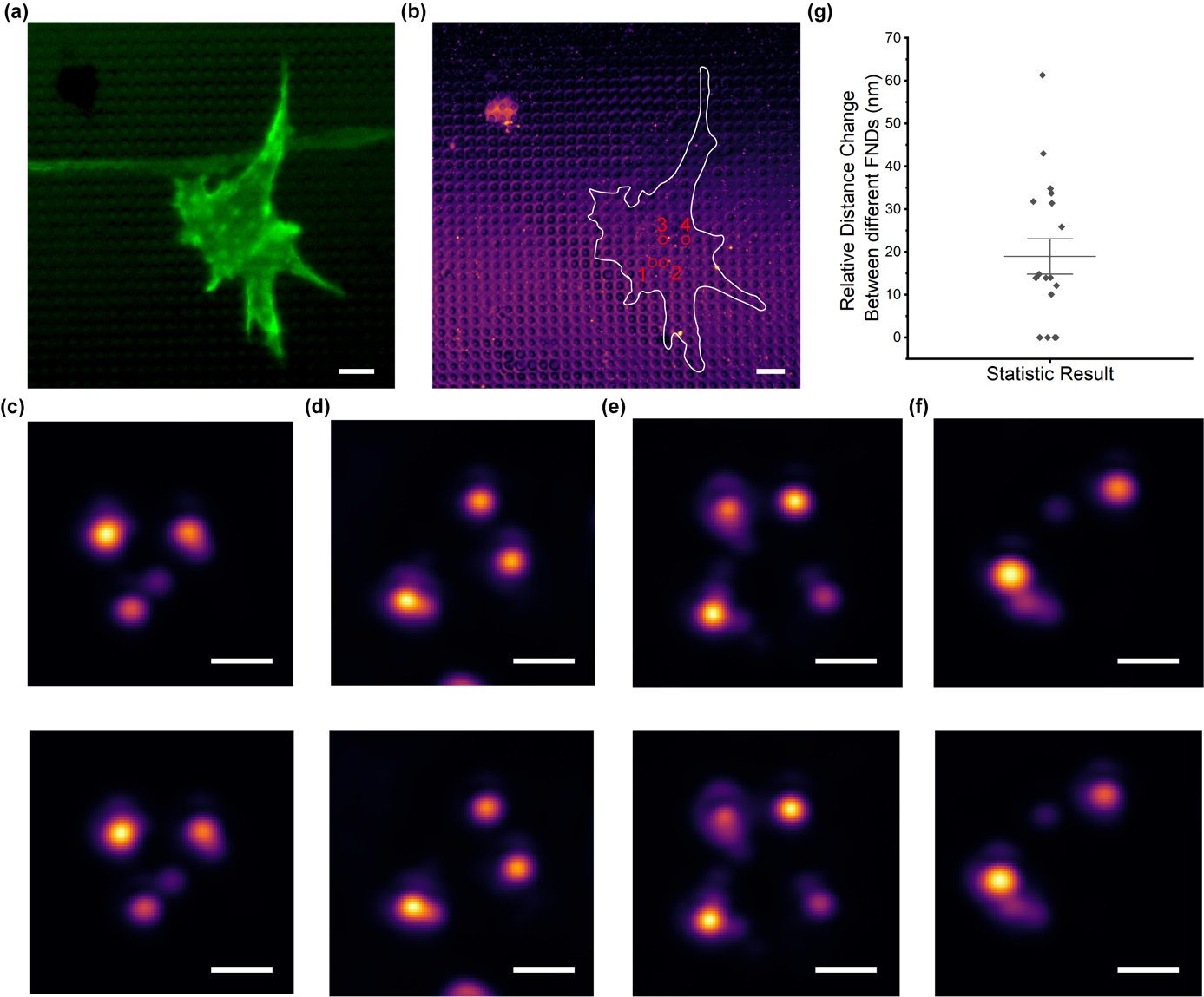


**Figure S5.** Stability test of the FND coating on PDMS micropillars. a) Fluorescence image of the NIH-3T3 cell used for the stability test. b) Confocal image of pillar tops located underneath the cell. The red line indicates the cell boundary. The scale bars in (a) and (b) are both 10 μm. c-f) Confocal images of selected pillar tops before and after cell lysis. The scale bars are 1 μm. g) Statistical analysis of the changes in relative distance between FNDs on four selected pillars.

To validate the stability of the FND coating on the PDMS micropillars, we used an upright confocal microscope to investigate the relative position change of FNDs attached to the pillar tops. **Figure S5(a)** shows the location of the NIH-3T3 cell analyzed in this experiment. Four pillars located underneath the cell were randomly selected, and the relative distances between FNDs on each pillar were measured before and after cell removal (**Figure S5(b-f)**). The changes in relative distance are summarized in **Figure S5(g)**. The average change in distance is 18.9 nm. These results indicate that the FND coating remained stable during the experiment, with no obvious evidence of nanodiamond detachment or slipping on the pillar surface.

**Optical properties and selection criteria of the FNDs**


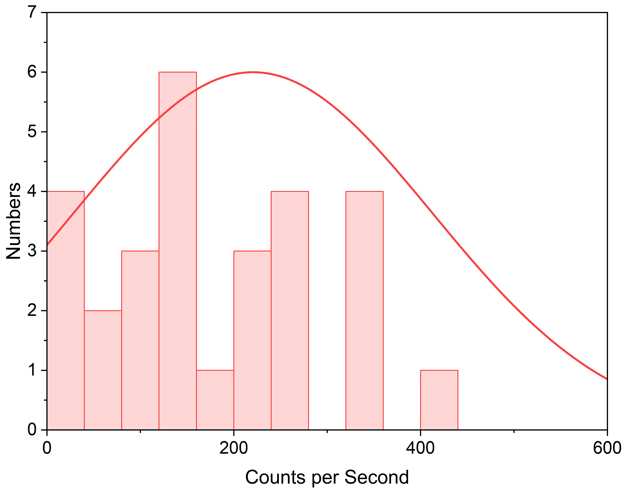


**Figure S6.** Statistics of the FND fluorescence intensity.

When selecting FNDs for the experiments, fluorescence intensity is the primary criterion. In general, the FND shows the highest fluorescence intensity on each pillar is selected. Based on our experimental experience, a fluorescence intensity above 1 Mcps satisfies the minimum signal requirement for measurements. Statistical analysis of 30 FNDs shows that 76.7% exceeded this threshold, as shown in **Figure S6.**

In addition to fluorescence intensity, the ODMR spectrum is used as a secondary selection criterion. FNDs exhibiting fewer than four pairs of ODMR peaks are preferred for further analysis. An ODMR spectrum with more than 4 pairs of peaks indicates signal overlap from multiple FNDs, preventing subsequent analysis.

ODMR contrast is another critical parameter in the selection process, as low ODMR contrast typically results in a poor signal-to-noise ratio in the subsequent LPM spectra. Therefore, FNDs with an ODMR contrast greater than 1% are selected. Based on the statistical analysis of 23 FNDs with fluorescence intensities above 1 Mcps, 78.3% also satisfied the ODMR contrast criterion.

**Supporting Information Note 3: Measurement Setup**


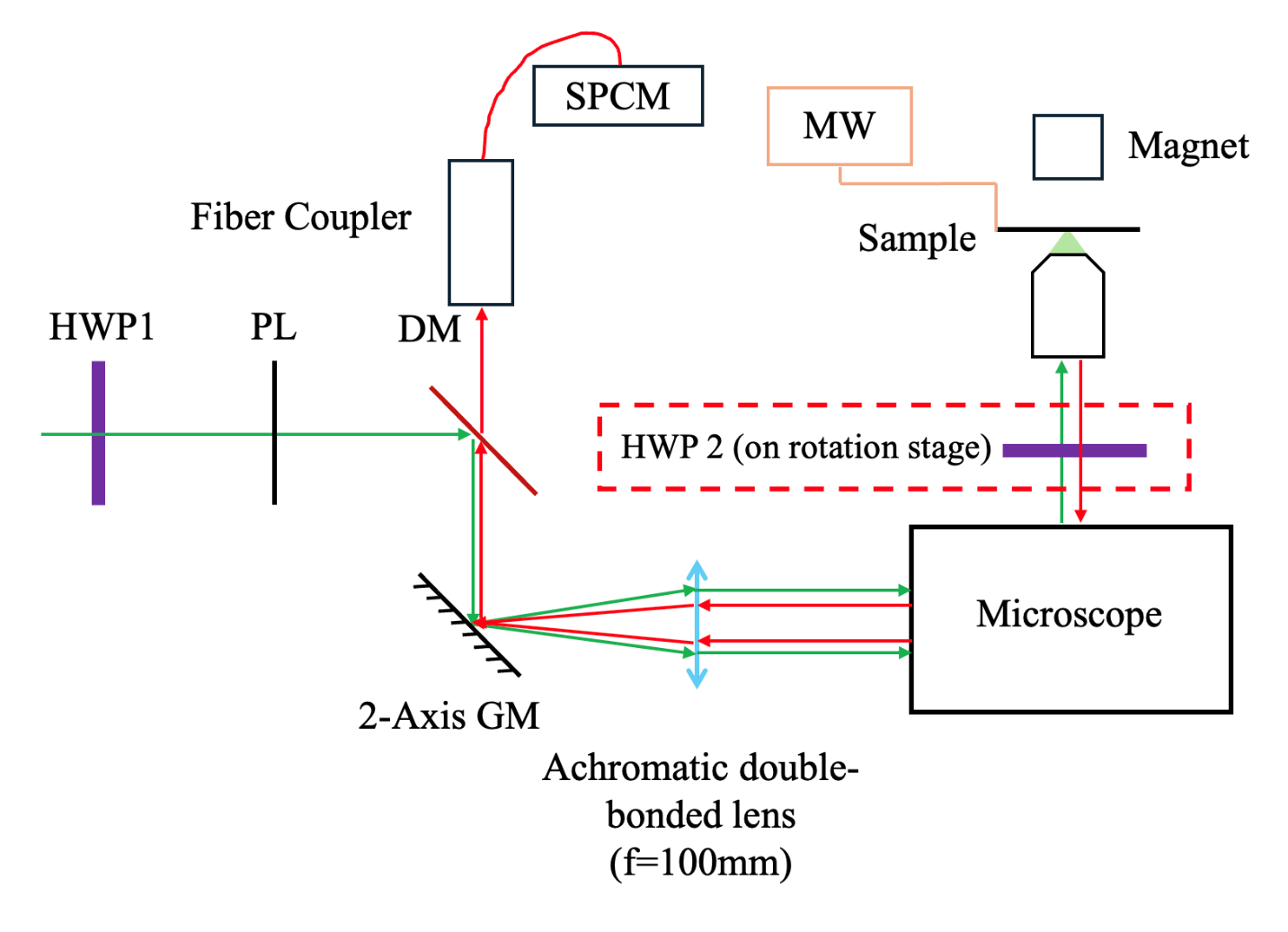


**Figure S7.** Schematic of the measurement setup. HWP: half-wave plate, PL: Polarizer, DM: dichroic mirror, GM: galvo mirror, SPCM: single photon counting module, MW: microwave.

The home-built confocal microscope system (shown in **Figure S7**) used in this study is based on a Nikon Ti2 microscope body. A 532 nm linearly polarized green laser serves as the excitation source. The laser first passes through a half-wave plate (HWP1; HWP25-532A-M, LBTEK) to adjust its polarization orientation. This adjustment is necessary because reflective optical elements can disturb the polarization state unless the incident angle is normal, or the incident is pure s- or p-polarized light ^[6]^. After that, the beam passes through a polarizer (PL; FLP25-VIS-M, LBTEK) to enhance the degree of polarization. A 560 nm long-pass dichroic mirror (DM; FF560-Di01-25x36, Semrock) is then used to separate the 532 nm excitation light from the NV center fluorescence, which primarily lies in the red spectral range (>637 nm). The laser beam is then directed onto a two-axis galvanometric mirror (GVS012, Thorlabs) for beam scanning. The galvo mirror is controlled via analog signals generated by a data acquisition card (PCIe-6321, National Instruments). An achromatic doublet lens (f = 100 mm, MAD508-A, LBTEK) is positioned before the side port of the microscope and serves as the first lens in a 4f optical system. Its front focal plane is aligned with the centers of two galvanometric mirrors, and its back focal plane coincides with the intermediate image plane of the microscope (typically where a camera sensor is placed). The second lens in the 4f system is the internal tube lens of the Nikon microscope, with a focal length of 200 mm. To manipulate the laser polarization, a second half-wave plate (HWP2; HWP25-532A-M, LBTEK) is mounted on a motorized rotation stage (EM-RP60, LBTEK) and placed between the tube lens and the objective. The excitation beam is then focused onto the sample via an air objective with a numerical aperture of 0.95 (UPLANXAPO40X, Olympus).

Fluorescence emitted by the sample is coupled into an optical fiber using a fiber coupler (MBT613D, Thorlabs) with a 10× objective (PLN10X/0.25, Olympus). The signal is detected by a single-photon counting module (SPCM-AQRH-44-FC, EXCELITAS), which is an avalanche photodiode (APD).

For spin manipulation, a microwave signal is generated by a microwave source (SynthNV Pro, Windfreak Technologies), amplified by a high-power amplifier (ZHL-16W-43-S+, Mini-Circuits), and delivered to the sample via an omega-shaped coplanar gold waveguide patterned on the cover glass.

An external magnetic field is applied to the sample using a permanent magnet placed above the objective lens.

**Supporting Information Note 4: Validation of the direction of the magnetic field**

To verify that the magnetic field applied by the permanent magnet was oriented along the vertical axis, we performed ODMR measurements on a bulk diamond with a [100]-cut. In such a crystal, the NV centers are theoretically oriented along four different body diagonals. If the external magnetic field is aligned vertically (as shown in **Figure S8a**), this geometry leads to only two resonance peaks in the ODMR spectrum. Using this criterion, we try to align the direction of the magnetic field.

After the alignment of the magnetic field, to further validate its direction, we rotated the bulk diamond horizontally and measured the ODMR spectra, fitting the measured ODMR spectrum with the method that we proposed before. The fitting result gives the $\beta$ and $\gamma$ angle. In theory, the $\beta$ should be $54.7^{\circ}$ and $\gamma$ should be $0^{\circ}$. The measured result shown in **Figure S8b** confirms that the magnetic field maintained vertical alignment.


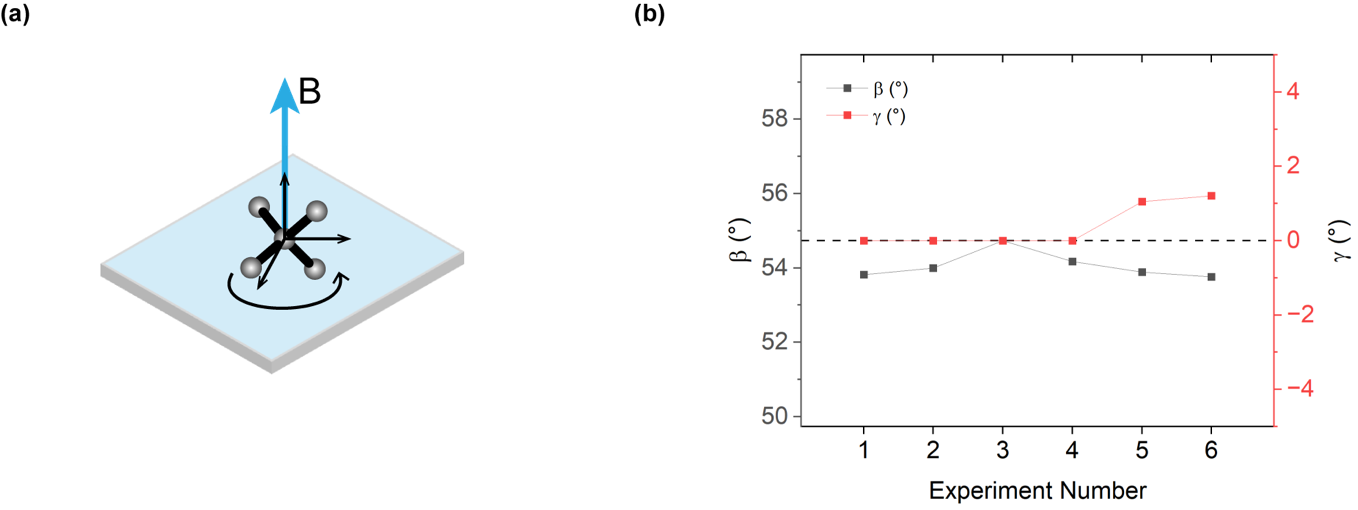


**Figure S8.** Validation of the magnetic field orientation. a) Schematic of the experiment setup. b) Fitting result of $\beta$ and $\gamma$. The horizontal dashed line indicates the theoretical value of $\beta$ and $\gamma$ when the direction of the magnetic field is perpendicular to the sample plane.

**Supporting Information Note 5: Relocation of the permanent magnet**

During the experiment, the permanent magnet sometimes needs to be moved away and then returned to its original position. Therefore, it was necessary to test the change in the magnetic field direction after relocating the permanent magnet. To investigate this, we selected a single FND and performed ODMR measurements after each magnet relocation. The experiment was repeated 10 times. In principle, only the angles $\beta$ and $\gamma$ obtained from the ODMR method are affected. As shown in **Figure S9**, the change in the measured angles is less than 1$^{\circ}$, and the standard deviations of the 10 measurements for $\beta$ and $\gamma$ are 0.10$^{\circ}$ and 0.34$^{\circ}$, respectively. This result indicates that the magnet returns to nearly the same position and that the magnetic field remains almost unchanged after relocation.


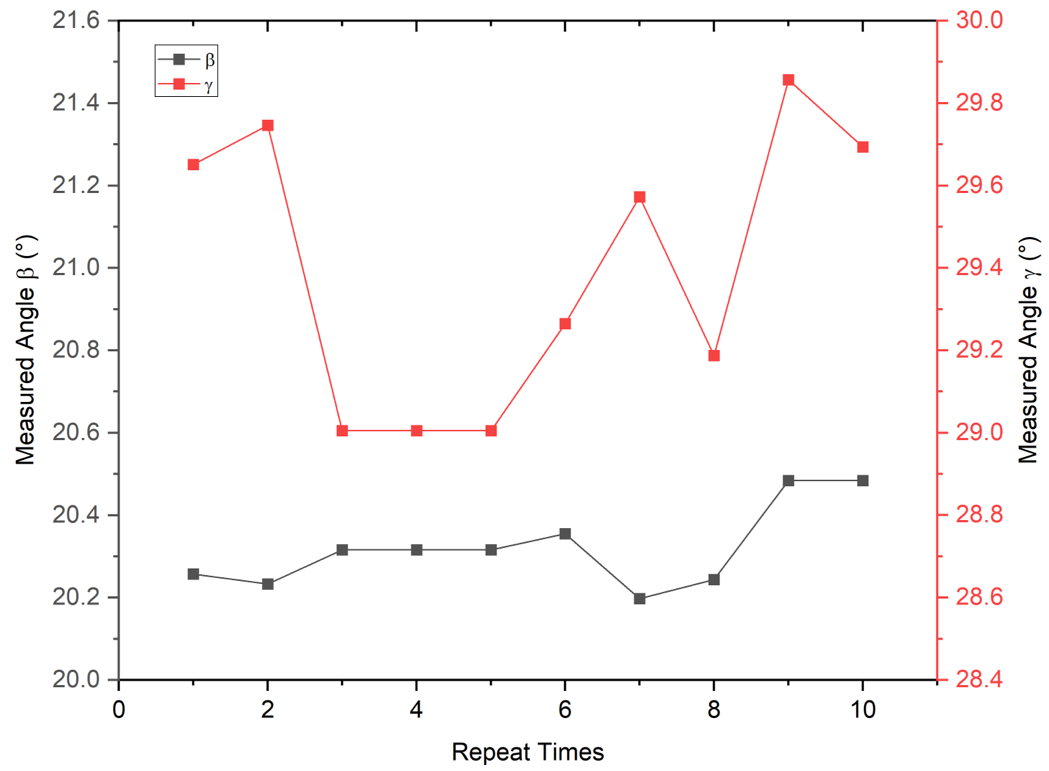


**Figure S9.** Measurement result of $\beta$ and $\gamma$ for the same FND after magnet relocation.

**Supporting Information Note 6: Theoretical Model for Determining NV Orientation via ODMR-LPM Hybrid Method**

**NV Orientation and Determination of Rotational Motion**


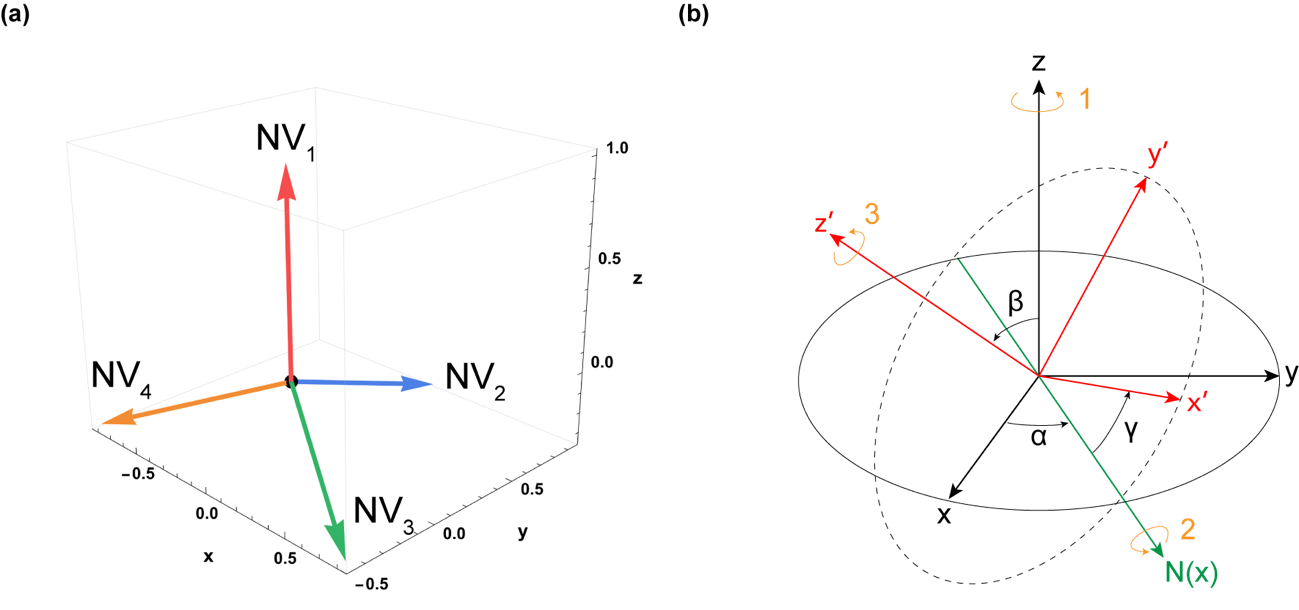


**Figure S10.** a) The four crystallographic orientations of NV centers in the reference state. b) The Euler angle following the ZXZ convention.

In a single-crystalline diamond, the NV center can adopt one of four crystallographic axes, corresponding to: $(111)$, $(\bar{1}\bar{1}1)$, $(\bar{1}1\bar{1})$, and $(1\bar{1}\bar{1})$, expressed in Miller indices. These four directions are symmetrically equivalent in the diamond lattice ^[7]^.

To fully determine the three-dimensional rotation of an FND, we track the relative orientations of the ensemble of NV centers inside. We define a reference diamond orientation ${NV}^{ref}$ (denoted as the reference frame) in which the $(111)$-oriented NV center is aligned with the laboratory Z-axis, and the $(\bar{1}\bar{1}1)$-oriented NV lies within the Y-Z plane (**Figure S10a**).

In this reference configuration, the unit vectors corresponding to the four NV axes are:

${NV}_{1}^{ref}=(0,0, 1)$, ${NV}_{2}^{ref}=\left( 0,\frac{2\sqrt{2}}{3},-\frac{1}{3} \right),$

${NV}_{3}^{ref}=(\frac{\sqrt{6}}{3},-\frac{\sqrt{2}}{3},-\frac{1}{3})$, ${NV}_{4}^{ref}=\left( \frac{\sqrt{6}}{3},-\frac{\sqrt{2}}{3},-\frac{1}{3} \right).$

The orientation of the diamond in the laboratory frame is parameterized using Euler angles $(\alpha,\beta,\gamma)$ following the ZXZ convention (shown in **Figure S10b**, all the following Euler angle follows ZXZ convention). This means the rotation is composed of three sequential steps:

1. A rotation by angle $\alpha$around the laboratory z-axis, yielding an intermediate frame $(x^{'}, y^{'}, z^{'})$,

2. A rotation by angle $\beta$ around the new $x^{'}$-axis, producing frame $(x^{''}, y^{''}, z^{''})$,

3. A final rotation by angle $\gamma$ around the new$z^{''}$-axis, resulting in the final orientation of the diamond.

The rotation matrix of the operation is^[8]^

Rotation about z-axis:

$$R_{z}\left( \theta\right)=\left( \begin{matrix} Cos(\theta) & -Sin(\theta) & 0 \\ Sin(\theta) & Cos(\theta) & 0 \\ 0 & 0 & 1 \end{matrix} \right)$$

Rotation about x-axis:

$$R_{x}\left( \theta\right)=\left( \begin{matrix} 1 & 0 & 0 \\ 0 & Cos(\theta) & -Sin(\theta) \\ 0 & Sin(\theta) & Cos(\theta) \end{matrix} \right)$$

The rotation matrix of the Euler angle $(\alpha,\beta,\gamma)$:

$$R(\alpha,\beta,\gamma)=R_{z}\left( \alpha\right)R_{x}\left( \beta\right)R_{z}\left( \gamma\right)$$

The corresponding NV orientation is:

$${NV}_{i}^{'}=R(\alpha,\beta,\gamma){NV}_{i}^{ref}$$

By applying this composite rotation to the reference NV vectors, one can compute the expected orientations of the NV centers under arbitrary diamond rotation. Because all these 4 NV axes are symmetrically equivalent in the diamond lattice, they can be randomly chosen.

Assume that the Euler angles of the diamond in two distinct orientations, denoted as state 1 and state 2, are given by $(\alpha_{1},\beta_{1},\gamma_{1})$ and $(\alpha_{2},\beta_{2},\gamma_{2})$ respectively. Let $R_{1}=R(\alpha_{1},\beta_{1},\gamma_{1})$and $R(\alpha_{2},\beta_{2},\gamma_{2})$ represent the corresponding rotation matrices constructed using the ZXZ Euler convention.

The relative rotation matrix $R_{rel}$, which describes the rotation of the diamond from state 1 to state 2, is given by:

$$R_{rel}=R_{2}\cdot R_{1}^{-1}$$

This matrix transforms vectors expressed in the reference frame of state 1 into their corresponding representations in state 2. It can be directly applied to the NV orientation vectors to compute their new orientations after the diamond rotation, thereby enabling precise determination of the diamond’s three-dimensional rotational motion.

Given the relative rotation matrix $R_{rel}$, the corresponding rotation axis and rotation angle can be extracted to provide an intuitive geometric interpretation of the diamond's reorientation.

The rotation angle $\theta$ is calculated using the trace of the rotation matrix ^[8]^:

$$\theta={Cos}^{-1}\left( \frac{Tr\left( R_{rel} \right)-1}{2} \right)$$

Where $Tr\left( R_{rel} \right)$ denotes the trace (the sum of the diagonal elements) of the rotation matrix.

The unit rotation axis $u=(u_{x},u_{y},u_{z})$ can be obtained from the off-diagonal elements of $R_{rel}$ as follows:

$$u_{x}=\frac{R_{32}-R_{23}}{2Sin\theta}$$

$$u_{y}=\frac{R_{13}-R_{31}}{2Sin\theta}$$

$$u_{z}=\frac{R_{21}-R_{12}}{2Sin\theta}$$

where $R_{ij}$ denotes the element in the $i$-th row and $j$-th column of $R_{rel}$. These expressions are valid when $\theta\neq0,\pi$. In degenerate cases (e.g., 0° or 180° rotation), axis extraction requires special handling; however, such cases are very rare in practice, so we omit the related discussion.

**Modeling of ODMR-LPM Hybrid Method for NV Axis Identification**

Here, we present the theoretical basis of using the ODMR-LPM hybrid method to determine the orientation of the NV axis and the corresponding Euler angles of the FND.


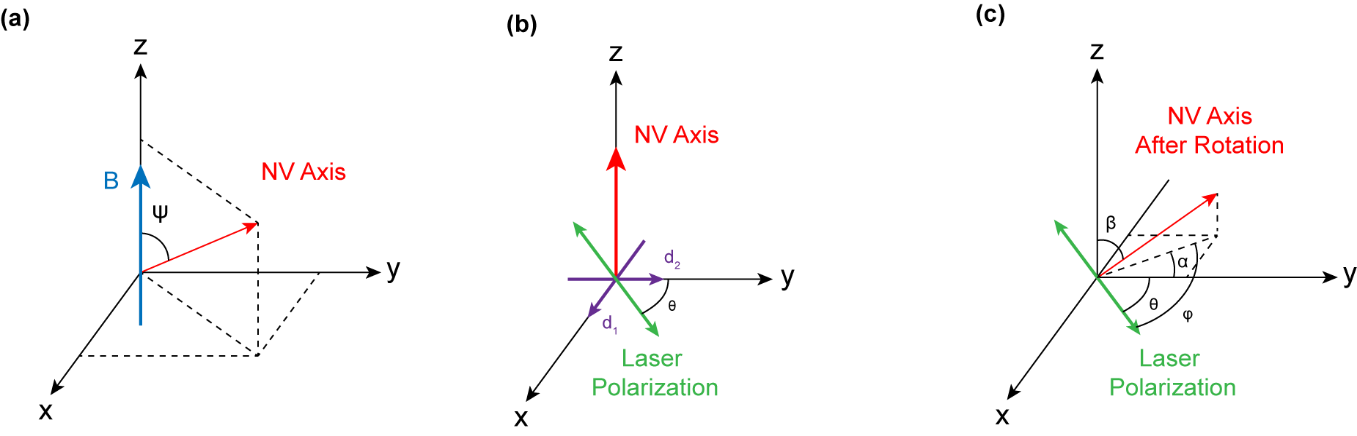


**Figure S11.** a) The angle between the NV axis and the magnetic field. b) Schematic of the NV axis and the corresponding dipoles. The laser polarization direction is also indicated in the figure. c) Schematic of the angle between the projection of the NV axis on the x-y plane and laser polarization direction after rotation.

The angle between the external magnetic field can be measured from the frequency shift of the ODMR spectrum ^[9]^. The ground state of the NV center is a spin triplet state. The Hamiltonian of it under an external magnetic field $\vec{\boldsymbol{B}}$ can be written as ^[10]^:

$$H=D{(\vec{NV}\cdot\hat{S})}^{2}+\gamma_{e}\vec{B}\cdot\hat{S}$$

Where $D\approx2.87 GHz$ is the zero-field splitting between the $m_{s}=0$ state and $m_{s}=\pm1$ states, $\hat{S}$ is the spin operator, and $\gamma=28 {MHz}/{mT}$ is the electron gyromagnetic ratio. The resonant frequency can be written as ^[9]^:

$$f^{\pm}=D+\frac{3\gamma_{e}^{2}B^{2}}{2D}{sin}^{2}\psi\pm\gamma_{e}Bcos\psi\sqrt{1+\frac{\gamma_{e}^{2}B^{2}}{{4D}^{2}}{tan}^{2}\psi{sin}^{2}\psi}$$

Where $\psi=arccos\frac{\vec{NV}\cdot\vec{B}}{B}$ is the angle between the NV axis and the extremal magnetic field. When the orientation and magnitude of the extremal magnetic field B are given, the angle $\psi$ can be fully determined by fitting the resonant frequency. In fact, in our experiment, because the magnetic field direction is vertical (the validation is shown in the SI), the calculated angle $\psi$ is the angle between the NV center and the Z-axis (as shown in **Figure S11a**). However, this formula also shows that the rotation around the z-axis can not be solved because it will not induce any change of the $\psi$. This can be solved by applying another method that can measure the horizontal component.

To resolve ambiguity in NV axis identification, we use an excitation–polarization–dependent fluorescence method. It is well-established that the excitation efficiency of an NV center is governed by the coupling strength between the electric field vector of the excitation laser and the dipole moments associated with the NV’s electron orbitals ^[11]^.

Each NV center possesses two orthogonal dipole vectors, $\vec{d_{1}}$ and $\vec{d_{2}}$, both lying in the plane perpendicular to the NV axis ^[12-14]^. The excitation probability for each dipole is proportional to the square of the projection of the laser’s electric field $\vec{\varepsilon}$ onto the dipole vector:

$$\Gamma\propto\left| \vec{\varepsilon}\cdot\vec{d} \right|^{2}$$

The linearly polarized laser has a polarization angle $\theta$ measured from the Y-axis (as shown in **Figure S11b**), and the corresponding electric field vector is expressed as:

$$\vec{\varepsilon}=E\left( \begin{matrix} sin\theta\\ cos\theta\\ 0 \end{matrix} \right)$$

Assuming an NV center aligned along the [001] crystallographic direction, its two dipoles in the initial configuration (before rotation) can be represented as ^[11, 13]^:

$$\vec{d_{1}}=d\left( \begin{matrix} 1 \\ 0 \\ 0 \end{matrix} \right), \vec{d_{2}}=d\left( \begin{matrix} 0 \\ 1 \\ 0 \end{matrix} \right)$$

After applying a general rotation parameterized by the Euler angles $(\alpha,\beta,\gamma)$ following the ZXZ convention, the rotated dipole vectors become:

$$\vec{d_{1}^{'}}=R_{z}\left( \alpha\right)R_{x}\left( \beta\right)R_{z}\left( \gamma\right)\cdot\vec{d_{1}}$$

$$\vec{d_{2}^{'}}=R_{z}\left( \alpha\right)R_{x}\left( \beta\right)R_{z}\left( \gamma\right)\cdot\vec{d_{2}}$$

Since the fluorescence intensity $I$ is proportional to the total excitation probability, it follows that:

$$I\propto\Gamma_{d_{1}^{'}}+\Gamma_{d_{2}^{'}}$$

$$\propto\left| \vec{\varepsilon}\cdot\vec{d_{1}^{'}} \right|^{2}+\left| \vec{\varepsilon}\cdot\vec{d_{2}^{'}} \right|^{2}$$

Under further simplification, the intensity can be expressed as:

$$I\propto dE(1-\sin^{2}\beta\cos^{2}\varphi)$$

$$I\propto1-\sin^{2}\beta\cos^{2}\varphi$$

where $\varphi=\alpha+\theta$. Here, as shown in **Figure S11c**, $\alpha$ represents the azimuthal angle of the NV axis in the laboratory frame (rotation around the Z-axis, counterclockwise), and $\theta$ is the polarization angle of the incident laser with respect to the Y-axis (clockwise). Therefore, $\varphi$ quantifies the angle between the laser’s polarization direction and the projection of the NV axis onto the transverse (xy) plane. Meanwhile, $\beta$ denotes the polar angle between the NV axis and the laser propagation direction (assumed to be the Z-axis).

This relationship reveals that the fluorescence intensity reaches a maximum when $\varphi=0$, corresponding to the situation in which the laser polarization is aligned with the projected NV axis. Thus, by measuring the intensity variation as a function of the laser polarization angle, the orientation of a single NV axis can be precisely determined.

However, in FNDs that contain multiple NV centers with different crystallographic orientations, the fluorescence signals from different NVs are superimposed. To resolve this, an external magnetic field can be applied to lift the degeneracy of the NV spin states ^[15]^. As a result, the ODMR frequencies of NV centers with different orientations are split due to the magnetic field's different projections along their respective axes.

Because microwave excitation induces spin transitions at specific resonant frequencies, the ODMR contrast (the difference in fluorescence intensity between microwave-on and microwave-off states) will be selectively modulated only by the NV center that is on resonance. Consequently, even in a multi-NV system, the angular dependence of the ODMR contrast at a given frequency reflects the orientation of a specific NV axis. In this way, the projection direction of an individual NV can still be determined.

**Fitting Method**

As established in the previous section, the orientation of any ND can be regarded as a rotation from a reference configuration orientation ${NV}^{ref}$, characterized by a set of Euler $\left( \alpha,\beta,\gamma\right)$.

Specifically, for ${NV}_{1}^{ref}$, we have:

$${NV}_{0}^{'}=R\left( \alpha,\beta,\gamma\right){NV}_{0}^{ref}=\left( \begin{matrix} sin\left( \alpha\right)sin\left( \beta\right) \\ -cos\left( \alpha\right)sin\left( \beta\right) \\ cos\left( \beta\right) \end{matrix} \right)$$

Based on this formula, we can tell its projection on the x-y plane forms an angle of $(\frac{\pi}{2}-\alpha)$, which is only related to $\alpha$, and the change in $\beta$ and $\gamma$ will not influence it. Based on this, by measuring its projection on x-y plane, the value of alpha can be fully determined. That can be measured using the LPM method.

The angle $\beta$ and $\gamma$ can be measured based on the ODMR method. In fact, because the Euler angle $\alpha$ represents the rotation around the Z-axis, the change of $\alpha$ will not cause any change in the ODMR spectrum. Based on this, we can split the horizontal and vertical rotation.

To determine the angle $\beta$ and $\gamma$, we directly fit the ODMR spectrum of the ND with the following function ^[10]^:

$$S\left( f \right)=c-\sum_{i} a_{i}\left[ \frac{{\Delta f}^{2}}{4{(f-f_{i}^{-})}^{2}+{\Delta f}^{2}}+\frac{{\Delta f}^{2}}{4{(f-f_{i}^{+})}^{2}+{\Delta f}^{2}} \right]$$

Where $c$ is the baseline, $a_{i}$ is the contrast of NV centers along the i-th direction, $\Delta f$ is the FWHM. The $f_{i}^{\pm}$ is determined from the formula ^[9]^:

$$f_{i}^{\pm}=D+\Delta D+\frac{3\gamma_{e}^{2}B^{2}}{2(D+\Delta D)}{sin}^{2}\psi_{i}\pm\gamma_{e}Bcos\psi_{i}\sqrt{1+\frac{\gamma_{e}^{2}B^{2}}{{4(D+\Delta D)}^{2}}{tan}^{2}\psi_{i} {sin}^{2}\psi_{i}}$$

Where $\Delta D$ is the shift of the zero-field splitting, and:

$$\psi_{i}=arccos\frac{\vec{{NV}_{i}}\cdot\vec{B}}{B}$$

$$\vec{{NV}_{i}}=R(\alpha,\beta,\gamma)\vec{{NV}_{i}^{ref}}$$

We use the least-square fitting method, and the fitting parameters are Euler angle $\beta$ and $\gamma$, the baseline $c$, the contrasts $a_{i}$, shift of the zero-field splitting $\Delta D$, and the FWHM $\Delta f$.

In this way, the Euler angle $\alpha,\beta$ and $\gamma$ can be fully determined. Then the rotation of the ND can be solved based on measurement result of the states.

**Supporting Information Note 7: Sensitivity of the rotation angle measurement**


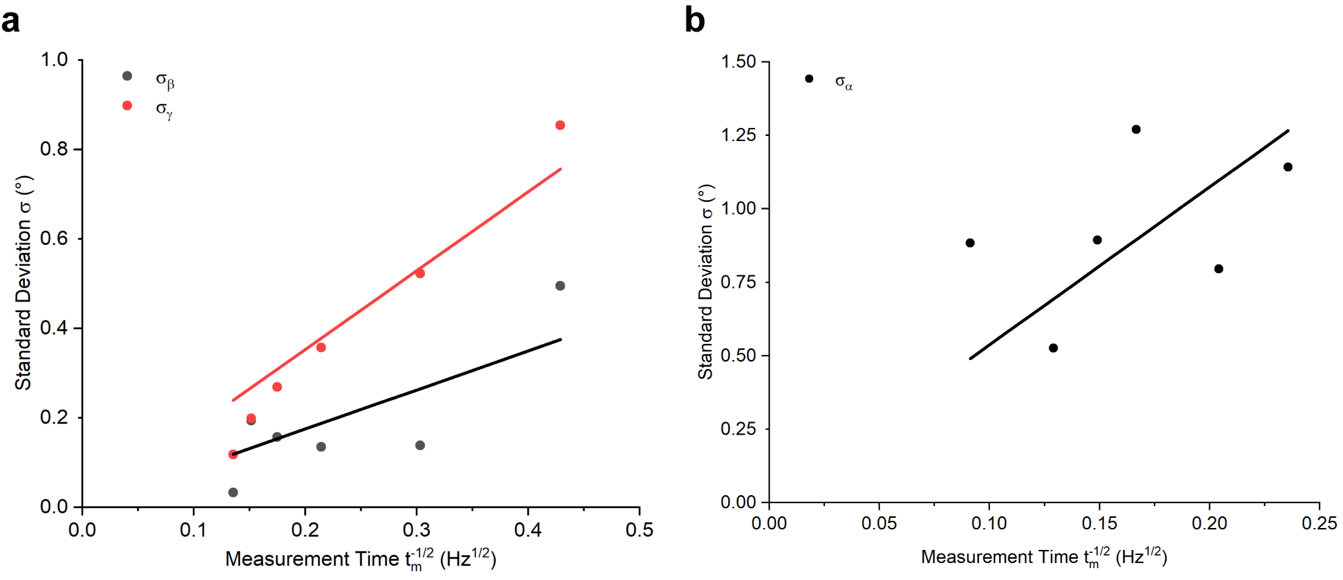


**Figure S12.** Standard deviation (STD) of the Euler angles as functions of the data acquisition time. **a.** STD of $\beta$ and $\gamma$ determined based on the ODMR-LPM hybrid method. The line is the linear fitting with 0 intercept. **b.** The STD of $\gamma$ determined based on the ODMR-LPM hybrid method.

We investigate the angle measurement sensitivity as shown in **Figure S12**. For the three Euler angles $\alpha$, $\beta$ and $\gamma$, $\alpha$ is determined from the LPM method, $\beta$ and $\gamma$ are determined from the ODMR method. To estimate the sensitivity, we measured the standard deviation of angles with different integration time. For $\beta$ and $\gamma$ getting from the ODMR method, we fit them with the shot noise function $\sigma=\frac{\eta}{\sqrt{t}}$. Their sensitivities are 0.87 $^{\circ} {Hz}^{-\frac{1}{2}}$ and 1.76 $^{\circ} {Hz}^{-\frac{1}{2}}$, respectively.

For $\alpha$ getting from the ODMR-LPM hybrid method, the result doesn’t show a clear linear relationship between the standard deviation and $t^{-\frac{1}{2}}$, which means that it is not shot noise limited. In fact, due to the measurement method, factors such as the stability of the turntable at different rotation speeds can also influence the measurement error. This may result in shot noise not accounting for the entire contribution to the total noise. The measurement standard deviation is around 1 degree.

|  | STD of $\alpha(^{\circ})$ | STD of $\beta(^{\circ})$ | STD of $\gamma(^{\circ})$ |
| --- | --- | --- | --- |
| FND 1 | 0.44 | 0.10 | 0.15 |
| FND 2 | 0.99 | 0.11 | 0.56 |
| FND 3 | 3.35 | 0.13 | 0.05 |
| FND 4 | 2.95 | 0.10 | 0.16 |
| FND 5 | 2.45 | 0.13 | 0.35 |

**Table S1.** Statistical results of the FND stability test.

We also measure the stability of FNDs attached to PDMS micropillars under physiologically relevant conditions (37 °C in cell culture medium). A total of 5 FNDs (fluorescence intensities ranging from 1.2 to 9.0 Mcps) are analyzed. The corresponding standard deviations of the measured $\alpha,\beta$ and $\gamma$ angle are shown in **Table S1**. The standard deviations remained within the measurement precision range (~3$^{\circ}$ for $\alpha$, which represents the in-plane angle, ~0.5$^{\circ}$ for $\beta$ and $\gamma$, which represent the out-of-plane angles).

**Supporting Information Note 8: Cytotoxic**


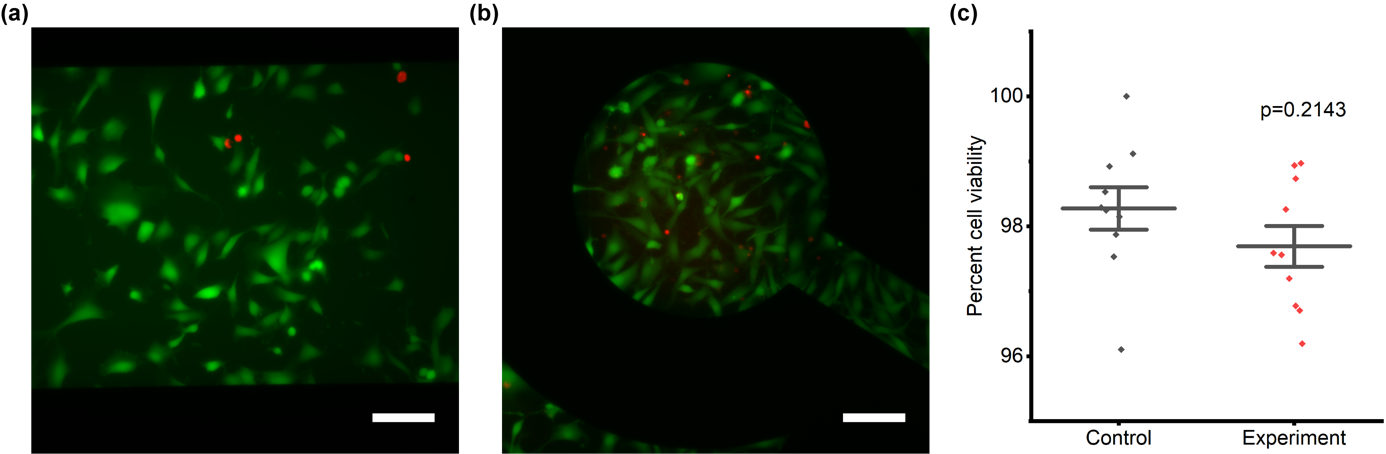


**Figure S13.** Cytotoxicity experiment results. a) Confocal image of the control group. b) Confocal image of the experimental group. c) Statistical analysis of cell viability. Cell viability remained above 95% in all groups. No statistically significant difference was observed between the control and experimental groups (t-test, p = 0.2143). The scale bars in (a) and (b) are 100 μm.

To evaluate the potential effects of laser and microwave, an additional cytotoxicity experiment was performed. The results are presented in **Figure S13**. Cell viability remained above 95% in all groups, and no statistically significant differences were observed between the exposed and control groups, indicating that the applied laser and microwave conditions did not induce measurable cytotoxicity. These results suggest that the proposed method has the potential to be applied to live-cell measurements without causing significant cell death.

**References**

[1] A. Öchsner, *Classical beam theories of structural mechanics*, Springer, **2021**.

[2] J. M. Gere, S. Timoshenko, Mechanics of Materials. ed, *Boston, MA: PWS* **1997**,

[3] I. Schoen, W. Hu, E. Klotzsch, V. Vogel, Probing cellular traction forces by micropillar arrays: contribution of substrate warping to pillar deflection, *Nano Lett* **2010**, 10, 1823.10.1021/nl100533c

[4] I. Schoen, Substrate-mediated crosstalk between elastic pillars, *Applied Physics Letters* **2010**, 97,

[5] K. Johnson, Contact Mechanics, Cambridge University Press, Cambridge, 1985, **1982**,

[6] G. Anzolin, A. Gardelein, M. Jofre, G. Molina-Terriza, M. W. Mitchell, Polarization change induced by a galvanometric optical scanner, *J. Opt. Soc. Am. A* **2010**, 27, 1946.10.1364/JOSAA.27.001946

[7] M. W. Doherty, N. B. Manson, P. Delaney, F. Jelezko, J. Wrachtrup, L. C. L. Hollenberg, The nitrogen-vacancy colour centre in diamond, *Physics Reports* **2013**, 528, 1.https://doi.org/10.1016/j.physrep.2013.02.001

[8] M. E. Rose, *Elementary Theory of Angular Momentum*, Wiley, **1957**.

[9] M. W. Doherty, J. Michl, F. Dolde, I. Jakobi, P. Neumann, N. B. Manson, J. Wrachtrup, Measuring the defect structure orientation of a single NV centre in diamond, *New Journal of Physics* **2014**, 16, 063067.Artn 063067

10.1088/1367-2630/16/6/063067

[10] L. Rondin, J. P. Tetienne, T. Hingant, J. F. Roch, P. Maletinsky, V. Jacques, Magnetometry with nitrogen-vacancy defects in diamond, *Rep Prog Phys* **2014**, 77, 056503.10.1088/0034-4885/77/5/056503

[11] T. P. M. Alegre, C. Santori, G. Medeiros-Ribeiro, R. G. Beausoleil, Polarization-selective excitation of nitrogen vacancy centers in diamond, *Physical Review B—Condensed Matter and Materials Physics* **2007**, 76, 165205

[12] M. W. Doherty, N. B. Manson, P. Delaney, L. C. L. Hollenberg, The negatively charged nitrogen-vacancy centre in diamond: the electronic solution, *New Journal of Physics* **2011**, 13.Artn 025019

10.1088/1367-2630/13/2/025019

[13] R. J. Epstein, F. M. Mendoza, Y. K. Kato, D. D. Awschalom, Anisotropic interactions of a single spin and dark-spin spectroscopy in diamond, *Nature Physics* **2005**, 1, 94.10.1038/nphys141

[14] V. R. Horowitz, B. J. Alemán, D. J. Christle, A. N. Cleland, D. D. Awschalom, Electron spin resonance of nitrogen-vacancy centers in optically trapped nanodiamonds, *Proceedings of the National Academy of Sciences* **2012**, 109, 13493.doi:10.1073/pnas.1211311109

[15] F. Münzhuber, F. Bayer, V. Marković, J. Brehm, J. Kleinlein, L. W. Molenkamp, T. Kiessling, Polarization-Assisted Vector Magnetometry with No Bias Field Using an Ensemble of Nitrogen-Vacancy Centers in Diamond, *Physical Review Applied* **2020**, 14, 014055.10.1103/PhysRevApplied.14.014055
